# Packet information encoding in a cerebellum-like circuit

**DOI:** 10.1371/journal.pone.0308146

**Published:** 2024-09-20

**Authors:** Alejo Rodríguez-Cattáneo, Ana Carolina Pereira, Pedro Anibal Aguilera, Ángel Ariel Caputi

**Affiliations:** Departamento de Neurociencias Integrativas y Computacionales, Instituto de Investigaciones Biológicas Clemente Estable, Montevideo, Uruguay; Georgia State University, UNITED STATES OF AMERICA

## Abstract

Packet information encoding of neural signals was proposed for vision about 50 years ago and has recently been revived as a plausible strategy generalizable to natural and artificial sensory systems. It involves discrete image segmentation controlled by feedback and the ability to store and compare packets of information. This article shows that neurons of the cerebellum-like electrosensory lobe (EL) of the electric fish *Gymnotus omarorum* use spike-count and spike-timing distribution as constitutive variables of packets of information that encode one-by-one the electrosensory images generated by a self-timed series of electric organ discharges (EODs). To evaluate this hypothesis, extracellular unitary activity was recorded from the centro-medial map of the EL. Units recorded in high-decerebrate preparations were classified into six types using hierarchical cluster analysis of post-EOD spiking histograms. Cross-correlation analysis indicated that each EOD strongly influences the unit firing probability within the next inter-EOD interval. Units of the same type were similarly located in the laminar organization of the EL and showed similar stimulus-specific changes in spike count and spike timing after the EOD when a metal object was moved close by, along the fish’s body parallel to the skin, or when the longitudinal impedance of a static cylindrical probe placed at the center of the receptive field was incremented in a stepwise manner in repetitive trials. These last experiments showed that spike-counts and the relative entropy, expressing a comparative measure of information before and after the step, were systematically increased with respect to a control in all unit types. The post-EOD spike-timing probability distribution and the relatively independent contribution of spike-timing and number to the content of information in the transmitted packet suggest that these are the constitutive image-encoding variables of the packets. Comparative analysis suggests that packet information transmission is a general principle for processing superposition images in cerebellum-like networks.

## Introduction

Understanding how the nervous system encodes and processes sensory images is still a challenging task for neuroscientists. The information on a given stimulus image encoded by a spiking pattern is strongly related to the discrepancy between the joint probability between stimulus image and spiking response and chance coincidence [1]. Stimuli and spikes occur over time, and timing is also involved while the information avalanche progresses through intricate networks. Thus, to discriminate novel stimulus patterns, and more importantly to identify and to attribute a proximal stimulus pattern to a given percept, it is necessary to preserve the time relationship between the spike distribution across the network and the timing of sensory relevant events. In addition, it is necessary to store patterns and, moreover, to be able to compare expected patterns and actual events. This understanding has led to the concept of packet information transmission in the visual system [2], and after being exhaustively tested and proven essential in artificial systems such as the internet [3], the same principle has recently been reviewed once again in seeking better general comprehension of information processing in biological neural networks [4].

A crucial step towards understanding how different neural networks process information is to recognize how specific neuron phenotypes establish specific functional connectivity within the circuit. This is because signals arising from every sensory receptor unit, while initially localized, then affect activity spread widely through the central nervous system [5]. In addition, individual neurons are in themselves complex integrative devices with intrinsic properties and morphology specifically adapted to the position and role that they have in the neural circuits [6]. Second order and higher order neurons project downstream in a rapidly divergent manner, but also have specific convergences on selected structures where different image features are decoded. This connectivity is entangled by feed-forward and feedback loops which cooperate in the construction of neural circuits whose dynamics can be both precise and complex [5–7].

Electric fish endowed with active electroreception [8] are an excellent choice to study how neural networks encode sensory images, since imaging strategies [9–11], peripheral encoding of sensory signals [12–14], and the anatomical structure of the early processing centers are accessible to experimental studies and are well known in many species [15–22]. In addition, neurophysical experiments combining individual neuron recordings with stimulus manipulation make it possible to inform the relationship between the association of activity pattern histories and the amount of information transferred [23–28]. An additional advantage is that evolutionary convergence producing similar forms of self-stimulation and signal processing have appeared in distant lineages, allowing comparative analysis of homologous and analogous systems [29, 30]. Active electroreception has evolved separately in African Gymnarchidae and Mormyrinae (both branches of Mormyroidea) and in American Gymnotiformes [29, 30]. Mormyrinae and pulse Gymnotiformes are so-called “pulse fish” whose electric organ discharges (EODs) consist of short pulses with a species-specific time course separated by silent intervals [8, 29–31], while Gymnarchidae and wave Gymnotiformes, so-called “wave fish’’ emit a sine-wave-like, continuous EOD [8, 29–31].

Thus, following the concept introduced by Arthur Krogh regarding the value of comparative studies [32], the EL of electric fish provides an exceptional neuroethological model to explore how sensory networks may process information and in which to compare differences and commonalities from different lineages and forms of reafferent input expressed in convergent evolution.

This article deals with central encoding of electric images in the electrosensory lobe (EL) of the pulse Gymnotiform *Gymnotus omarorum*. The EL is a bilateral, topographically organized anatomical structure which contains two paths, one consisting of a single layer of neurons, the so called fast electrosensory pathway whose function has been reviewed elsewhere [33–35], and a second, slow pathway which is a cerebellum-like network used for imaging the near environment. Information processing in this second, slow pathway is the focus of this report [24, 36–42].

**Fig 1:**
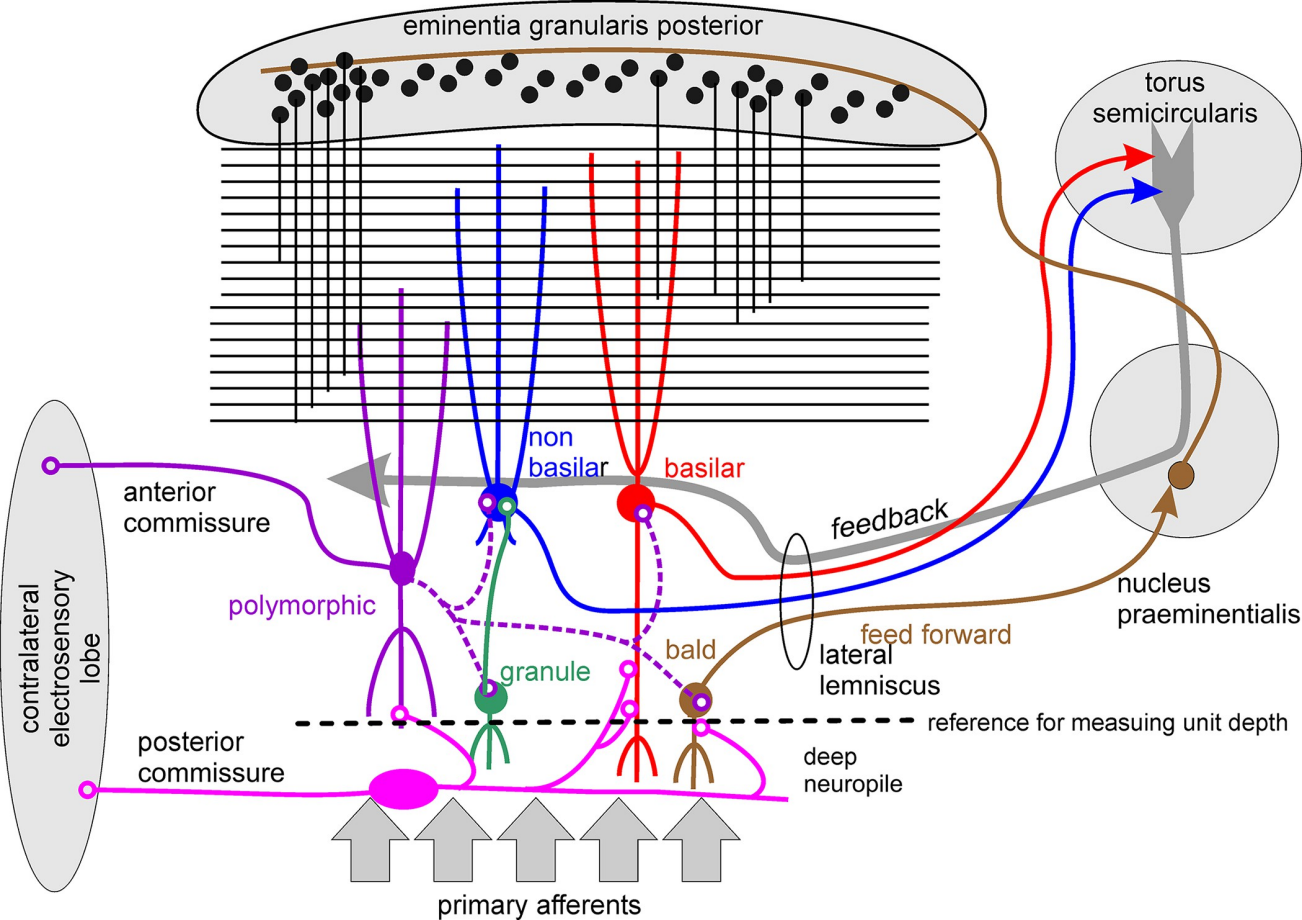
The main neuronal components of the EL in the context of the rhombencephalic circuit of the slow electrosensory pathway. The inputs of this circuit are the primary afferent terminals (gray arrows) in the deep neuropil layer, where they contact bald (brown), basilar pyramidal (red), granule (green), and polymorphic neurons (violet). They also contact oval (not shown) and multipolar neurons (magenta). Commissural connections between the ELs are carried by three neuron types, causing strong reciprocal inhibition: Multipolar and oval (not represented) neurons, scattered deeply in each EL, project to the contralateral EL through a posterior commissure. Polymorphic neurons (violet), whose somata are spread through the plexiform layer and show extensive basilar and apical dendritic processes, project through the anterior commissure. These neurons may also have inhibitory actions on pyramidal, granule, and bald neurons (dotted lines, violet lines). The output originating from the so-called “inner loop” is mainly carried by bald (brown) neurons located in the granule cell layer and perhaps some branches of the axons of superficial pyramidal neurons (red and blue). These axons project through the lateral lemniscus to the praeminentialis nuclei (brown path). Various praeminentialis cell types receive these inputs from these axons and project to the granule neurons of the eminentia granularis posterior, which in turn give rise to the parallel fibers in the EL molecular layer (black lines) that drive directly or through local interneurons the apical dendrites of the pyramidal output neurons. The main output of the EL and also of this “inner loop” are basilar (red) and non-basilar (blue) pyramidal neurons, which project to the torus semicircularis. Pyramidal neurons also receive recurrent localized feedback through an “outer loop” that includes the torus semicircularis and a different group of neurons in the praeminentialis nuclei (thick gray arrow).

The slow pathway component of the EL is the cornerstone of a rhombencephalic circuit for processing the electrosensory image flow. The circuit is similar to that of homologous ELs that have been well-described in wave Gymnotiformes [19, 20, 36–38, 41] (Fig 1).

The input of this circuit is a pattern of primary afferent spike bursts driven by every self-generated electric image [43, 44]. The main output of the EL and also of the whole rhombencephalic circuit is carried by two types of pyramidal neurons called basilar and non-basilar [42]. Each EL makes cross-connections with the contralateral EL by means of: a) two inhibitory neuron types (oval and multipolar) scattered in the deep layers and projecting through the posterior commissure and b) polymorphic neurons located in the granule and plexiform layers which project through the anterior commissure [45, 46]. The output projecting to the praeminentialis nuclei is mainly carried by bald neurons and perhaps also some branches of the axons of pyramidal neurons [41]. This projection is the origin of a functional feed forward loop which involves a praemientialis projection on the eminentia granularis posterior. The axons of granule neurons of eminentia granularis form a parallel fiber bus directly exciting and indirectly inhibiting, via stellate and ventral molecular layer inhibitory interneurons, the apical dendritic tree of pyramidal output neurons, [47–51] (not shown in the schemata of Fig 1). The EL receives at least two additional, descending inputs: one through the lateral lemniscus completing a feedback loop that projects directly or indirectly to the proximal dendritic tree of pyramidal neurons [21, 38, 50], and another from the raphe nuclei which provide a broadly distributed serotoninergic input EL [52].

In *G*. *omarorum*, different EL neurons fire either phase-locked, one-to-one with the EOD or in a phase-preferred way after the EOD [39, 41]. Theoretical constructs [53] and experimental evidence [28, 41] indicate that the EL compares the input carried by each discrete EOD with a moving average of the electrosensory inputs resulting from previous EODs. Here we expand the database of previously published natural responses to the EOD in drug free, decerebrated preparations focusing on the centro-medial pisciculi of the EL. Units were classified using hierarchical cluster analysis according to their timing after the EOD. This classification was cross-validated with two independent criteria: a) unit distance above the deep neuropil layer of the EL; b) the consistency of their responsiveness to stimulus objects. Our findings indicate that: a) the post-EOD spike timing pattern is a characteristic of the neuron type and therefore reflects the sub-threshold post-synaptic activation pattern; b) unit locations and firing patterns suggest that two of these unit types may correspond to the output pyramidal neurons; c) this pulse fish encodes each EOD generated image as a packet of unit activity where spike timing after the EOD and the spike number per EOD vary in a characteristic way for each neuron type and stimulus image. We discuss the value and generality of combining spike timing and spike number in constituting an information packet for efficiently encoding discrete sensory images, either self-generated or having a well-defined onset.

## Materials and methods

Nine specimens of the pulse species *Gymnotus omarorum* (15–25 cm length, undetermined sex) gathered in the Laguna del Cisne, Maldonado, Uruguay (geo-reference 34° 50’ 24.8" S, 55° 08’ 43.0" W) were used in this study. Experiments were performed under the accepted bioethical guidelines of international academic societies and approved under the protocol number 001/03/2011 of the animal care committee of the Instituto de Investigaciones Biológicas Clemente Estable, Montevideo, Uruguay.

The experiment consisted of extracellular unit recordings from the brain stem of decerebrate preparations while electrosensory stimuli were manipulated in freely discharging and respiring fish, in the absence of anesthesia (see details below). At the end of the experiments animals were euthanized with pentobarbital (10 mg, i/m).

### Surgery and decerebration

Decerebration consisted of complete removal of the forebrain which created a preparation without the possibility to integrate pain. This did not affect the brainstem control of either the EOD command or the peripheral generation and primary afferent encoding of electrosensory images, or the sensory integrative function of the EL.

In preparation for surgery, fish were immersed in progressively cooled aquarium water bubbled with carbogen (95% O2–5% CO2) [54]. At about 4–6°C fish stopped discharging and did not respond to pressure on the tail. At this point the fish was moved to a cellulose sponge holder immersed in the recording chamber (a 6 cm deep, 26 x 45 cm tank filled with aquarium water at 10°C, conductivity 100 μS/cm), where surgery was performed.

Under this deep anesthesia, the skin over the cranium was removed and two small holes were made on either side of the skull above the forebrain. The tip of a microprobe was introduced through one of the holes and the forebrain was aspirated while allowing air to penetrate through the other hole. This procedure sectioned the connection between the telencephalon and the diencephalon, leaving the optic tectum and more caudal brainstem intact.

After decerebration, in the absence of the forebrain, surgery could continue without pain. A 150 μm diameter, nichrome wire was passed through the two holes in the skull used to decerebrate the fish and was firmly attached to a holder screwed to the recording tank. To maintain the fish body in a straight position and under the water with the exception of the recording window above the EL, a cotton thread was passed lengthways through the dorsal muscular mass and attached to the caudal extremity of the holder supporting the fish. A small piece of the skull and the underlying meninges were removed to expose the EL, thus forming the recording window which was maintained above the water level.

### Electrophysiological recordings

To allow complete recovery from the cold anesthesia, recordings were started 4 hours after the end of surgery. By this time all fish had recovered their natural EOD rate and waveform. Novelty responses were provoked by changes in electrosensory images, and field potentials and unit activity showed clear electrosensory modulation.

In 5 fish, unit activity was recorded in the EL using a Michigan probe with 16 vertically aligned active sites separated by 50 μm. This configuration allowed us to position the recording sites in register with the laminar structure of the EL using field potentials previously characterized in detail as a reference [41]. Once the typical field potential of the deep neuropil layer was identified, we were able to evaluate the position of the unit in the layered structure by noting the distance from the best recording electrode to the deep neuropil layer for each unit, with a resolution of 50 μm. In another 4 fish, unit activity was recorded with paired tetrode arrays separated by 200 micrometers (Neuronexus, Ann Arbor, MI, USA), each bearing two 4-point active sites vertically separated by 200 micrometers. An indifferent electrode was placed in the cisterna magna in all cases.

Probe signals were multiplexed with a differential amplifier (AM systems 3600). Gain and filter settings were adjusted during the experiment according to spike waveform and signal to noise ratio (gain between 5000 and 20000, band-pass filtering more often at 300 Hz—5 kHz, for recording units, 10 Hz—5 kHz for recording field potentials). In all experiments two wires located on opposite sides of the tank were used to monitor the timing of the EOD through an additional differential amplifier (1800, AM Systems, USA, band pass, 300–5000 Hz, gain 100). Signals were simultaneously digitized at a sampling frequency of at least 20 kHz per channel (DataWave, SciWorks 8.0, Loveland, USA).

### Estimating unit positions within the EL

For 39 of the 102 units we were able to determine the distance between the best recording point for the unit and the deep neuropil layer where the electrode tip was located. To evaluate whether the different clusters identified were indeed located in different layers of the EL, the locations of the units belonging to each cluster were compared using a Kruskal-Wallis analysis of variance; this was followed by pairwise ranksum tests for evaluating differences in unit type locations within the EL with a significance level less than 0.05 according to the Holm-Bonferroni’s procedure.

### Spike transformation into point processes

Commercial routines (Experimenter DataWave, Loveland, USA) were employed for recording and on- and off-line separation of spikes. In the case of the units recorded using the 16 vertically aligned electrodes the signals recorded from each channel were first digitally subtracted from the mean of the waveforms recorded at the two nearest neighbor channels. This procedure allowed us to remove noise and determine in which layer of the EL the recorded units were situated.

In all experiments we separated and displayed putative spikes on-line. We used a manually-defined threshold to identify spikes. Spike peaks were tagged in the next millisecond after crossing the threshold and their timing relative to the EOD was noted. To distinguish different units, 2 ms epochs were selected around the tagged peaks and the Datawave Experimenter software used a digital amplitude and time window to identify and classify the different units. The time stamps of these selected units were displayed in rasters on-line during the experiments.

In a second view off-line, activity falling within a -2.5 to 2.5 ms window around the positive peak of the EOD was blanked in order to avoid the large artifact that it causes. Outside this blocked-out window, records were analyzed a second time applying the same procedure for selecting the 2 ms epochs around positive spike peaks; spike waveforms were sorted in clusters taking into account 3 to 6 parameters (peak to peak amplitude, interval between peaks, positive amplitude, negative amplitude, positive to negative ratio, ratio between negative peaks if it applies, Datawave Experimenter software). These parameters were plotted pairwise for each channel and waveform-shape clusters were manually defined taking into account the consistency of waveforms in all channels. Only units having waveform-shape clusters clearly non-intersecting with those of other units were considered in this study.

Once point processes of spikes and EODs were defined, their time series were analyzed in detail off-line using in-house Octave/Matlab routines (Supplementary material). We studied the baseline response to the EOD in 102 units, and of these, further analysis examined the changes in responses of the units to moving objects (19 units), and the changes in the responses of the units to increments in stimulus amplitude at the center of the receptive field (43 units).

### Analysis of baseline responses to the EOD

To evaluate the effect of the EOD on the spike trains in the absence of objects (other than the recording setup) cross-correlation histograms were calculated, providing an estimation of the probability of spike timing relative to the EOD, for individual units. It is important to note that the interval -2.5 to 2.5 ms was blanked to avoid the EOD artifact.

Peri EOD histograms with analysis time encompassing about 10 other EODs (i.e. a rough estimation of the cross-correlation) were estimated as follows: for each EOD of the recorded sample, we subtracted the timing of all spikes occurring in the period 200 ms before, and 300 ms after that EOD from the time stamp of such EOD. Next, we calculated the relative frequency distribution before and after the EOD. For each EOD we divided the analysis time (v.g. 200 ms before to 300 ms after each EOD) into consecutive 1 ms bins (bin zero is centered at each EOD) and counted the number of spikes in each bin. Then, the number of spikes firing in each bin number of all EODs was summated, divided by the total number of considered EODs, and multiplied times 100.

### Classification of unit types using hierarchical cluster analysis

To classify the units according to the immediate effects of the EOD on spiking behavior, a hierarchical dendrogram was constructed from bottom to top levels (i.e. from post-EOD histograms of individual units to that of the population). This analysis was based on 102 units in which the recordings obtained in the absence of objects were long enough to contain at least 500 EODs and 100 spikes. First, to evaluate spike probability distributions independently of the spike and EOD rate, we selected the period from 2.5 to 40 ms following the reference EOD in the cross-correlation histograms, and constructed post-EOD histograms normalized to the total number of spikes in the same 2.5–40 ms period in all considered EOD cycles. Second, we calculated the Euclidean distances between the bin counts of these histograms for every pair of units. Third, the triangular dissimilarity matrix composed of all possible distances was transformed as a vector and Ward’s procedure was used to generate clusters having minimum within-cluster variance. Fourth, we used different distance thresholds defining dendrogram cut-off levels to evaluate for each level the ratios between minimum inter-cluster and maximum intra-cluster distances (Davies-Bouldin index [55]). This ratio was plotted as a function of the number of resulting clusters for each threshold. This plot revealed a hyperbolic function whose incremental difference showed a clear elbow at about 6 clusters. Finally, to characterize each cluster with an image, the period -15 to 40 ms in each cross-correlation histogram for every unit was used to construct peri-EOD histograms. The mean, median, and extreme quintiles of the distribution of relative frequencies in each bin were calculated and considered as representative profiles of the post-EOD activation pattern of the units in each cluster.

### Exploration of spike firing probability with a moving object

For 19 units the stimulus consisted of moving a vertically oriented copper rod, (0.5 cm diameter, 4 cm tall) along the fish’s body parallel to the skin, at a distance of about 1 mm and at low speed (1 mm/s), from the foveal region to beyond the gills. Stimulus position was driven and recorded using a computer controlled Hewlet-Packard X/Y plotter. It is important to remark here that while these experiments inform on the differential responses of unit clusters to the same stimuli, and although this procedure is used in most studies on active electroreception, this stereotyped form of stimulation is merely representative. The true extent of any unit’s ‘receptive field’ is a complex concept since the presence of an object at any point in the near environment around the fish will restructure the electric field generated by the EOD and thus the electric image [9, 10]. A full, detailed exploration of the receptive field of each unit type, defined as the region of space where the presence or movement of an object causes a change in the unit response to the EOD, would imply the movement of objects of various shapes, sizes and materials, at different speeds in all directions in the volume surrounding the fish, which is beyond the scope of this article.

### Exploration of spike firing responses manipulating a local stimuli

Another 43 units were tested using a probe object consisting of a 1 cm long plastic cylinder with carbon electrodes at either end, placed perpendicular to the skin at the site where movements and changes in the resistance between the carbon electrodes caused the largest variations in firing rate. This was identified as the ‘center’ of the receptive field. Transitions between low and high intensity stimuli were produced by switching the longitudinal resistance of the object probe from 2.5 MΩ to 1kΩ, or vice versa. The effects of stimulus increases were evaluated using duty cycles of either 28s at 2.5 MΩ followed by 2s at 1 kΩ, or 23s at 2.5 MΩ followed by 7s at 1 kΩ. The effects of stimulus decreases were evaluated using duty cycles of either 28s at 1 kΩ followed by 2s at 2.5MΩ, or 20s at 1kΩ followed by 10s at 2.5MΩ. To study the steady-state effects we compared the results obtained in the initial periods of the two types of experiments. The voltage across the probe stimulating the skin was simultaneously monitored (AM Systems 1800 differential amplifier, USA, band pass, 10–5000 Hz, gain 100) and digitized.

We analyzed the effects of the increasing or decreasing stimulation regimes on spike number per EOD, and on spike timing distribution, separately. Each experiment consisted of several trials in which the stimulus was incremented in a stepwise manner, using a computer-controlled reed relay switch. From each of the trials we selected the responses to 25 EODs before and 25 EODs after the increment in the stimulus and numbered each EOD from EOD_-25_ to EOD_+25_. Then, we pooled the time-stamps of all the spikes evoked after each ordinal EOD from EOD_-25_ to EOD_25_, EOD_0_ being the first EOD that saw the stimulus change. Thus following each ordinal EOD we obtained the spike pattern distribution. Thirty-two units were studied systematically in experiments in which at least 16 trials of increasing and decreasing increments in object conductance could be performed.

To evaluate spike number per EOD, for each unit we summed the number of spikes evoked by each ordinal EOD in successive trials t. Then, for the units of each cluster, we calculated the mean spike number and the standard deviation per ordinal EOD from EOD_-25_ to EOD_25_. Differences in EOD-evoked spike numbers were first tested in control conditions (presumably in steady state conditions since the stimulus was constant during 20s of more), with either a high stimulus intensity, or a low stimulus intensity, using Friedman ANOVA test with 25 repetitions corresponding to the 25 EODs before a stimulus step in either direction. To test whether there was any systematic change in spike number per EOD when the stimulus was increased, for units of each cluster, we used a Friedman test based on 3 repetitions corresponding to the 3 EODs before and 3 EODs after the step increase. A similar procedure was used to test if there was any systematic change in spike number per EOD when the stimulus was decreased.

To test whether there was a change in spike-timing distribution, we first considered the pooled distributions of spike-timings from the responses to all ordinal EODs from: a) EOD_-25_ to EOD_-4_ (control distributions)_,_ b) EOD_-3_ to EOD_-1_ (immediate pre-step distributions)_,_ c) EOD_0_ to EOD_2_ (immediate post-step distributions), d) EOD_23_ to EOD_25_ (late post-step distributions). We evaluated the responses in the stimulus separately, according to the direction of the stimulus change: from increase to decrease, or vice versa, in the protocols defined above. We normalized each distribution for the number of spikes and then calculated the mean relative entropy (i.e. the difference in information) using the Jensen-Shannon divergence between a) control distributions before increases and before decreases of the stimulus, b) immediate pre-step and control distributions before increases in stimulus, and c) immediate pre- and control distributions before decreases in stimulus, d) immediate pre- and immediate post- step distributions before and after increases of the stimulus and e) immediate pre- and immediate post-step distributions before and after decreases of the stimulus, f) immediate post-step and late post-step distributions after increases of the stimulus and g) immediate post-step and late post-step distributions after decreases of the stimulus. In this context, the Jensen Shannon divergence may be interpreted as the mutual information between the binary variable defining two post EOD specific distributions and the distribution resulting from their average. To test the hypothesis that a step increment would increase the difference in information to a greater extent than the variation in information content observed when comparing two control distributions (control and immediate pre-step) evoked by the same EOD amplitude after a long-lasting period of steady stimulation, we used one sided signrank tests followed by Holm-Bonferroni’s procedure for correcting significance level. To evaluate whether the difference in information between two distributions is similar to the difference in information carried by the spike counts of such distributions we correlated the Jensen Shannon divergence with either the ratio between spike counts or the absolute difference in spike counts.

## Results

### Basal responses of the phase-preferring units to the EOD

In the EL of *Gymnotus omarorum*, the slow electrosensory pathway has two major unit types defined by their firing patterns in relation to the EOD: units that are phase-locked with the EOD, and phase preferring units which fire only sparsely, with slight timing variability and not in every inter EOD interval, but always with a similar timing distribution with one to three modes in specific phases of the inter-EOD cycle, where histogram relative peak amplitudes depend on the particular stimulus image [27, 28, 39, 41]. This article focuses on 108 “phase-preferring units” recorded extracellularly in the centro-medial map of the EL, which receives primary afferent input from the head projection region. Recordings of unit activity were obtained from 9 decerebrated fish, breathing spontaneously and with an EOD rate within the range normally observed in intact, freely behaving animals (mean: 44.5 ms equivalent to 22.5 EOD/s).

Basal responses were evaluated in the absence of objects, for 102 units whose recordings lasted at least 500 EODs. Many units fired action potentials sparsely, although statistically clearly related to the EOD: overall, the average inter-spike interval was 83.9 ms and the average number of spikes per EOD was 0.35 (grand mean across units).

Raster displays synchronized with the EOD suggested that in the different types identified, spiking probability after every discrete EOD has two components: one represented by the probability of firing a particular number of spikes after each EOD, a variable here referred to as spike number, and the second represented by the distribution of preferred times after the EOD in which a given neuron type fires, a variable referred to as spike-timing.

**Fig 2:**
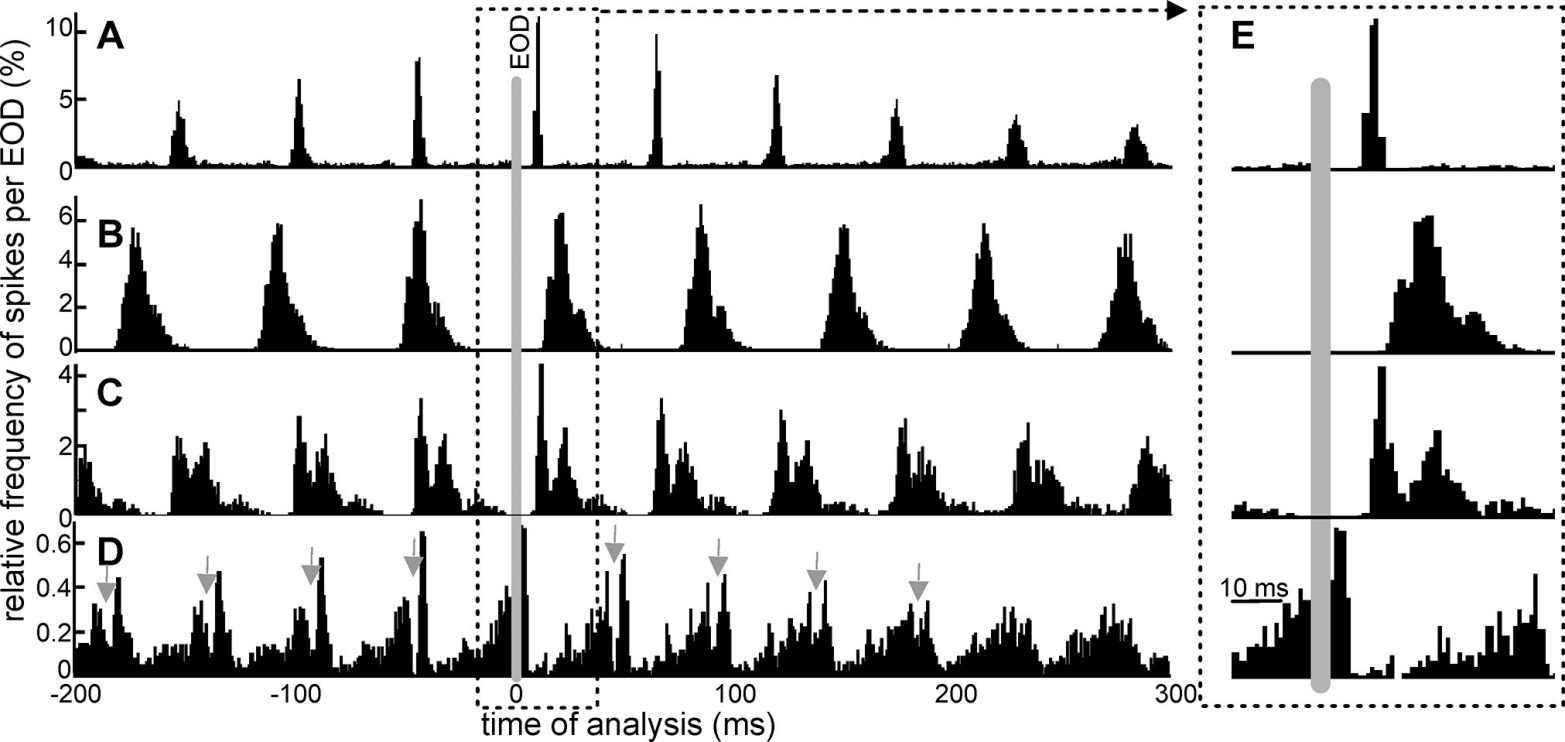
Cross-correlation histograms suggest that phase preference firing is characteristic of the unit. A-D) Cross-correlation histograms show the relative frequency of firing a spike within the previous 200 ms and next 300 ms after each EOD (gray bar at time 0 corresponds to the reference EOD; bin width: 1 ms). In D, gray arrows indicate a notch artifact resulting from blanking the EOD. E shows a time-expanded version of the peri-EOD histograms (dashed line bordered rectangles) illustrating differences in the timing distributions between units. While units in A–C are mainly excited by the EOD, the unit in D is mainly inhibited.

The cross-correlation histograms relating spike timings to the EOD emission over the 500-EOD-long series suggests that units can be classified according to their post EOD firing pattern histograms. We were able to clearly separate 6 different unit types distinguished by their characteristic firing patterns in response to the EOD-provoked reafferent electrosensory input. Fig 2 illustrates four examples. The unit illustrated in Fig 2A shows the sharpest recruitment, where spikes occurred between 10 and 12 ms after the EOD. Fig 2B shows a unit with a longer and more variable latency, giving a broader post-EOD distribution with a peak about 22 ms after the EOD. Fig 2C illustrates a third type of unit with a bimodal, repetitive post EOD pattern. For the unit type shown in Fig 2D firing probability reaches a maximum in the 5 ms immediately after every EOD, followed by an abrupt, marked reduction in spiking and then a smooth increase in firing probability with time leading up to, and immediately after the next EOD (Fig 2E bottom) This remarkable post-EOD, phase-related, reduction in spiking indicates the presence of a strong inhibition driven by the EOD. Note that the deep gap in the rising flank of the cross-correlation histogram (marked by arrows in Fig 2D) is an artifact due to the EOD blanking window (see methods).

For the purpose of unit classification, the 102 units were characterized by their post-EOD spike-timing histograms (1 ms bins, spanning from 2.5 to 40 ms following the EOD, respectively). To focus on the spike timing pattern, post-EOD histograms were normalized by the total number of spikes within each inter-EOD interval. Two-sample Kolmogorov-Smirnov-tests contrasting the 5151 possible pairs of post-EOD distributions were calculated. Ninety, 87, or 83 percent of the comparisons respectively indicate significant differences at Holm-Bonferroni adjusted p-values lower than 0.05, 0.01 and 0.001. This suggests that the firing patterns are characteristic of different neuron functional types. To evaluate this possibility, hierarchical cluster analysis was used to group the post-EOD histograms according to the Euclidean distances between them.

**Fig 3:**
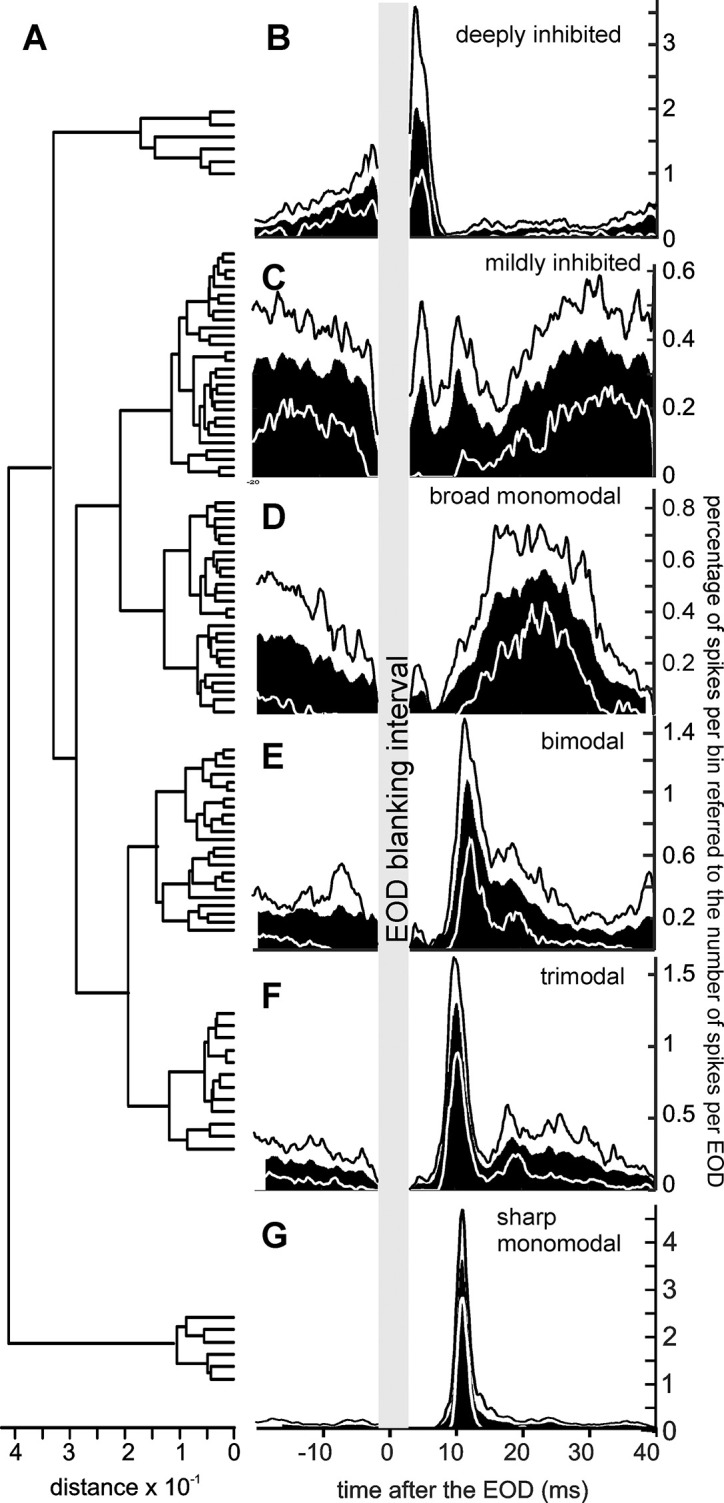
Unit classification. A) Dendrogram representing the Euclidean (Ward’s method) dissimilarity between post-EOD histograms normalized to the number of spikes. B-G) Representative profiles of the six major clusters selected, taking into account the Davies-Boulin index. These profiles correspond to the median (black area) of the peri-EOD histograms and the 20 (white line) and 80 (black line) spike frequency percentiles. Units were classified according to their profiles as follows: B) Deeply inhibited units are sharply inhibited at about 5 ms after the EOD, followed by a slow increase in firing probability continuing through the EOD blanking period up to 5 ms after the next EOD; C) Mildly inhibited units, have their maximum firing probability either immediately after the EOD or at the end of the inter-EOD interval, showing one or two brief reduced firing periods at 5 and 10 ms after the EOD; D) Broad monomodal units fire one or two spikes, with a variable latency 10 to 30 ms after the EOD and a broad histogram with a mode about 23 ms; E) Bimodal units firing either sharply at about 10–12 ms after the EOD, broadly between 15 and 25 ms or two spikes, one in each of these windows; F) Trimodal units fire within the same sharp window as in E but have a broader late firing probability shoulder, and also an occasionally recruited spike firing sharply at 5 ms; G) Sharp monomodal units are silenced by the EOD, fire sharply at 10–12 ms, followed by sporadic firing over the rest of the inter-EOD interval.

Fig 3A shows the dendrogram obtained using Ward’s procedure. Panels B-G show the 20, 50 and 80 percentile values of the peri-EOD histograms corresponding to the 6 best separable clusters obtained after considering the Davies-Bouldin index [54], calculated as the ratio of minimal inter-cluster distance against maximal intra-cluster distance. In Fig 3B–3G, the parallelism of the profiles at the 20 (white), 50 (histogram) and 80 (black) percentile values illustrates the similarity of the post EOD spike-timing patterns characteristic within each cluster. This led us to name the unit types by their distinct firing patterns: deep inhibited (Fig 3B), mildly inhibited (Fig 3C), broad monomodal (Fig 3D), bimodal (Fig 3E), trimodal (Fig 3F), and sharp monomodal (Fig 3G), according to the shape of their peri-EOD histograms.

The results of this cluster analysis suggest that these identifiable firing behaviors are the product of specific synaptic activation sequences targeting neurons with distinct phenotypes, positions, and roles in the EL circuit [40]. As described in the following sections, two corollaries of this hypothesis were tested experimentally: a) recording depths were found to be characteristic for the units belonging to each cluster; b) the peri-EOD firing patterns of the neurons belonging to each cluster were similarly altered by changes in the electrosensory images.

#### Unit types belong to different EL layers

**Fig 4:**
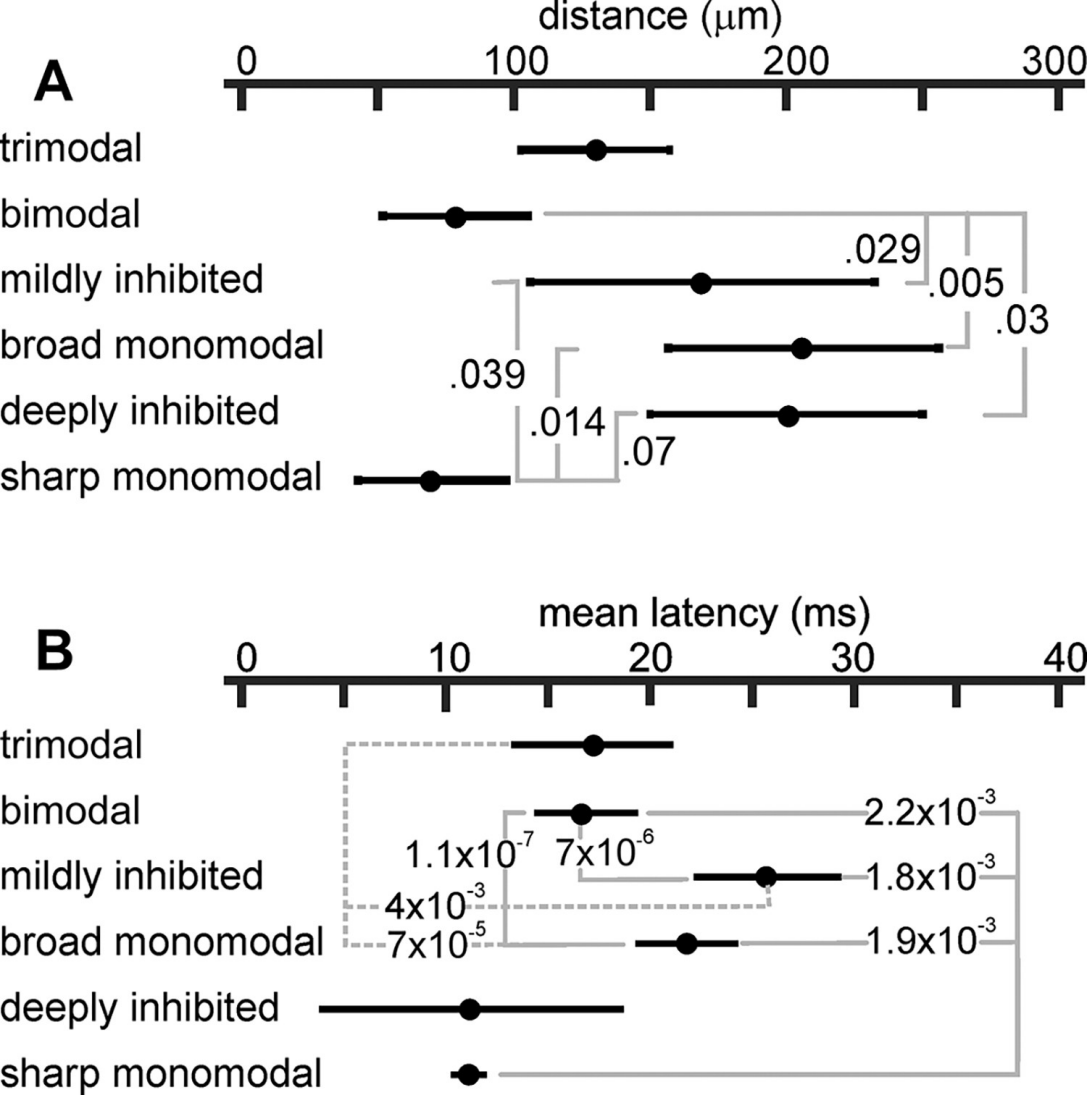
Different unit types were recorded in different EL layers. A) The plot shows the mean and standard deviation of the unit distances to the deep neuropil layer. The Kruskal-Wallis test (χ2 = 26.2, df = (5, 43), p = 8x10-5), followed by multiple pairwise ranksum tests, indicated that distances depend on unit type. Holm-Bonferroni corrected p-values are: bimodal vs. broad monomodal (0.0047); sharp monomodal vs. broad monomodal (0.0218); bimodal vs. mildly inhibited (0.0289); bimodal vs. deeply inhibited (0.0303); sharp monomodal vs. mildly inhibited (0.0385); sharp monomodal vs. deeply inhibited (0.0794). This indicates that sharp monomodal and bimodal units are deeper in EL than broad monomodal, deeply inhibited, and mildly inhibited units. B) For the different unit types, the plot shows the mean spike latency and standard deviation. The Kruskal-Wallis test (χ2 = 67.9, df = (5, 102), p = 2.7x10-13), followed by multiple pairwise ranksum tests, indicated that spike firing time was characteristic of unit type. Holm-Bonferroni corrected p-values are: bimodal vs. broad monomodal (1.1x10-7); bimodal vs. mildly inhibited (7x10-06); trimodal vs. broad monomodal (7x10-6); sharp monomodal vs. mildly inhibited (0.0018); sharp monomodal vs. broad monomodal (0.0019); bimodal vs. sharp monomodal (0.0022); trimodal vs. mildly inhibited (0.0029); broad monomodal vs. mildly inhibited (0.004). This indicates that sharp monomodal, bimodal, and trimodal units fire earlier than broad monomodal and mildly inhibited units.

Bimodal and sharp mono-modal unit types were recorded respectively at 78 ± 26 μm (mean ± standard deviation, N = 7) and 70 ± 27 μm (N = 6) above the probe tip, significantly deeper in the EL than the position of mildly inhibited (206 ± 49 μm, N = 8) and broad monomodal (169 ± 63 μm, N = 13) units. Bimodal units were also significantly deeper than deeply inhibited units, situated at 200 ± 50 μm (N = 5). Fig 4A illustrates these data and p-significant values of post-hoc multiple pairwise rank sum tests, compensated for multiple comparisons using Holm-Bonferroni criteria. Although the pairwise rank sum test between these deeply inhibited and sharp monomodal units yielded a p-value of 0.0079, Holm-Bonferroni’s criterion of significance was not satisfied, probably due to the large number of comparisons. Trimodal units were found at intermediate locations (130 ± 30 μm, N = 4), not significantly different from any of the other clusters.

In the light of the known anatomy [21, 37, 41], these results suggest that sharp mono-modal and bimodal units are located at about the granule cell layer, while the broad monomodal, mildly inhibited, and deeply inhibited units correspond to the firing of the more superficial layers containing pyramidal neurons.

#### Unit types have different mean latencies but similar basal rates

In the EL of wave-emitting Gymnotiformes, there is a robust correlation between baseline firing rate, somata location, and the extension of the apical dendritic tree [20, 50, 51]. However, our data from the pulse Gymnotiform *G*. *omarorum* did not verify this observation. When contrasting the number of spikes per EOD generated by units recorded at different locations, no significant differences were found (Kruskal-Wallis tests: χ2 = 5.84, df = 5, p = 0.21). Neither was a difference found when the number of spikes per EOD was compared between the units belonging to different clusters (χ2 = 2.93, df = 5, p = 0.569, respectively). Instead, and also differently from wave-emitting Gymnotiformes, clear differences were found in the post-EOD spiking patterns, as is indicated by the mean latency values (Fig 4B). Deeply located types fired significantly earlier than superficially located neurons. Overall, this analysis suggests that while in *G*. *omarorum* the functional differences of deep and superficial neurons are expressed in their latencies and spiking patterns, in wave fish the main differences between superficial and deep layer neurons are expressed in spike number per second [50, 51].

### Characteristic responses of unit types to a moving metal object

**Fig 5:**
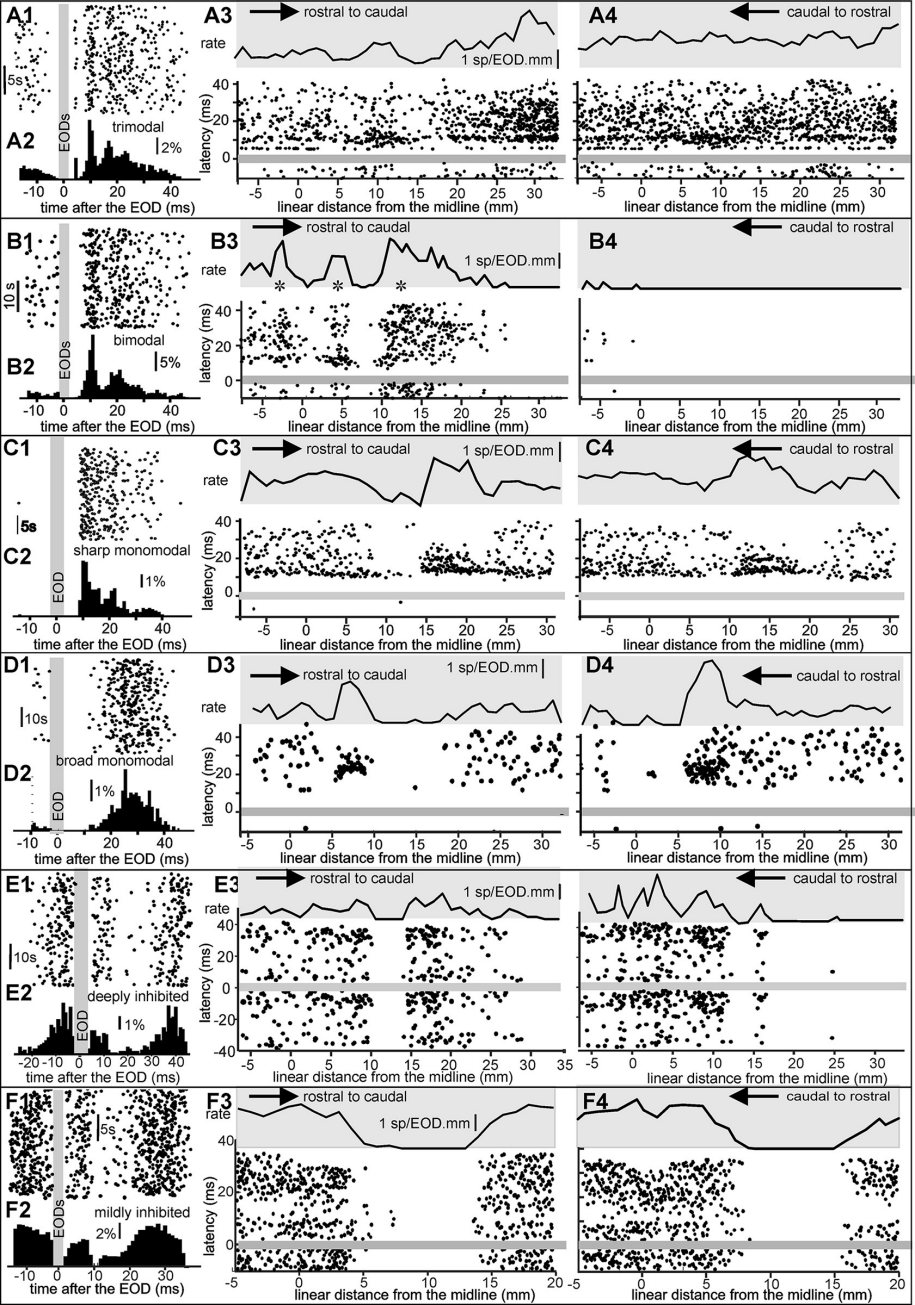
Spike timing modulation by a moving object within the receptive field. Panels A to F correspond to distinct unit types: trimodal, bimodal, sharp monomodal, broad monomodal, deeply inhibited, and mildly inhibited. Each panel shows, at the left, the peri-EOD raster (subindex 1) and its corresponding peri-EOD histogram (subindex 2) in the absence of objects. The right panels show the rasters when moving a vertical copper rod 1mm parallel to the skin, rostral to caudal from the snout to the operculum (subindex 3), and caudal to rostral (subindex 4) at a speed of 1 mm/s. In the raster diagrams, the gray bars indicate the EOD-blanking period; the arrows indicate the direction of movement. The black curve on the gray background above the raster represents the spike number per EOD, calculated as the number of spikes divided by the number of EODs for every millimeter along the trajectory (the bottom limit of the gray background indicates the absence of spikes).

In a first series of experiments, changes in the reafferent electric images were created by moving a copper rod (the stimulus object: 5 mm diameter, 3 cm length, vertically oriented) back and forth from the snout to the gills, parallel to the skin at a distance of 1 mm from the electroreceptive surface, at 1 mm/s. Although these experiments were not designed to make a complete exploration of the receptive field, the object evoked clear changes in the number of spikes per EOD, and in spike timing distributions, in 20 units. In the other 16 units the modulations, either in spike number per EODs or spike-timing distribution, were not sufficient to be clearly identifiable. The rasters of peri-EOD spike timings and the histograms corresponding to one example from each unit type are shown in Fig 5A–5F.

The upper traces in Fig 5A3–5F3 show the spike firing rate as a function of the distance from the fish’s buccal midline, as the copper rod was moved rostral to caudal, parallel to the electroreceptive skin surface. The raster diagram shown below shows spike timing before and after successive EODs. The six panels illustrate three general phenomena found in all unit clusters: a) Opposing patterns of spike firing and b) opposing patterns of spike number per EOD were observed for adjacent sites on the skin, giving an indication of the center of the receptive field for each of the different units; and c) different patterns of spike firing and number of spikes per EOD were observed for the same sites when the object was moved in opposite direction indicating movement direction sensitivity of the network.

For example, in a trimodal unit (Fig 5A3, object moving in rostral to caudal direction), when the stimulus object was at 10 mm from the buccal midline there was a relative maximum spike number per EOD and, in particular, a higher spike density at about 10 ms after the EOD. When the stimulus object was on either side of this point, spike number per EOD was lower and the spike latencies were more dispersed. In the same unit an early spike, firing relatively frequently at a fixed latency of 5 ms from -7 to 3 ms reduced their firing probability in the region where the peak probabilities at 10 ms increased and when the object was moved in the opposite direction (Fig 5A4, caudal to rostral) there was no clear change in rate but there was a clear change in the spiking pattern.

Bimodal units, for example the unit illustrated in Fig 5B, in the absence of any object showed their characteristic pattern. They were inhibited just after the EOD and showed a sharp peak at 10 ms and a broader peak at about 20 ms. They also showed center surround opposition and movement direction sensitivity. Two out of these recorded bimodal units exhibited the largest changes in response pattern when the object was moved rostral to caudal or caudal to rostral. As the stimulus object moved over the skin surface, going from rostral to caudal (Fig 5B3), firing activity showed a “double surround”, spatially complex response, alternating increased firing probability (centered on positions 3 ms, 5 ms and 10–15 ms) with increased inhibition centered on positions 20–40 mm and 60–90 mm. When the same object was moved over the same skin region in the opposite direction (Fig B4), the unit was essentially silenced, illustrating a strongly increased, widespread inhibition.

A simpler, inhibition-excitation-inhibition, center-surround, profile was observed for the sharp monomodal unit of Fig 5C, where again, the modulation pattern depended on the direction of object movement. Another unit of this type showed mild inhibition when moving from rostral to caudal and no change when moving from caudal to rostral.

**Fig 6:**
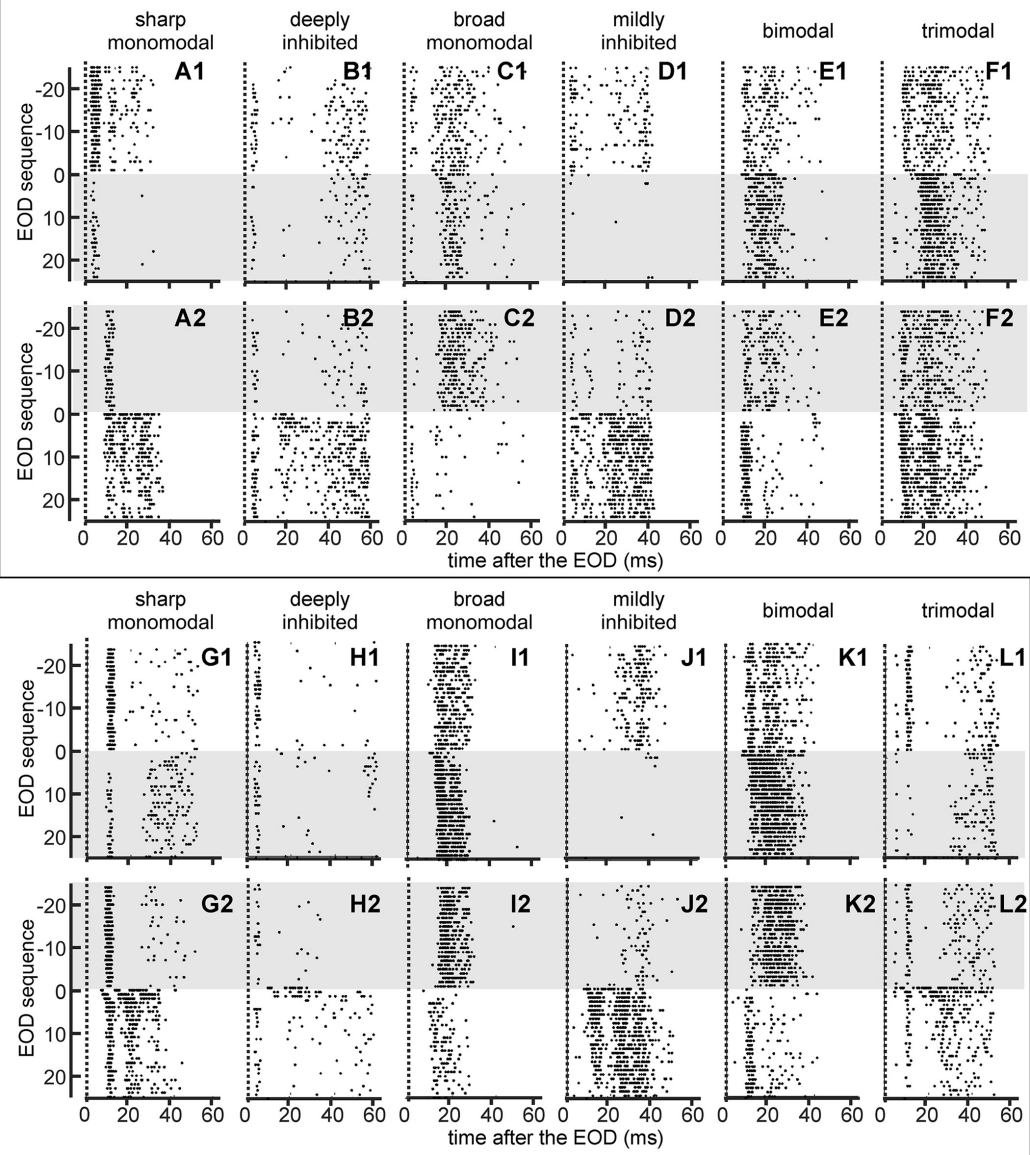
The effects of stepwise changes in stimulus intensity on spike timing. Rasters of two typical units belonging to each unit type are illustrated in the upper (A to F) and lower (G to L) panels, respectively. Repeated trials are plotted superimposed. Each point corresponds to the spike timing after each of the 25 EODs which occurred before, and the 25 EODs after a step change in stimulus intensity. The increment in object conductance was adjusted to produce a 3-fold increase (subindex 1) or decrease (subindex 2) in stimulus amplitude. Unit types are: A and G) sharp monomodal; B and H) deeply inhibited; C and I) broad monomodal; D and J) mildly inhibited; E and K) bimodal; and F and L) trimodal. Gray backgrounds correspond to strong stimuli, and white backgrounds correspond to weak stimuli. Vertical dotted lines at zero indicate the timing of the EOD.

Interestingly, sharp monomodal units fired a burst in the regions where the object provoked increased excitation (Fig 5C3–5C4). The units of this type also fired a burst when the impedance of a stationary object was decreased (see Fig 6, below) suggesting that this bursting response is correlated with the decrease in stimulus.

The three other unit types which are located more superficially in the EL showed “Mexican-hat-like” center-surround patterns when the object was moved over the skin. The region of maximal effect (referred to as the “center” of the receptive field) varied in extension for different units. The complexity of these ‘receptive fields’ is also illustrated by the shift in the absolute position of the ‘center-surround’ profile when the object moved in opposing directions: the regions of maximal effect were displaced in a direction opposite to the object movement.

For broad monomodal units, at the center the moving object increased the spike firing rate and reduced latency dispersion (Fig 5D, N = 4). In these neurons the surround effects after the object movement left the center region were more marked than when the object approached the maximal responsiveness region, suggesting a sensory filter for detecting the direction of movement (Fig 5D).

An opposite center-surround pattern was seen for mildly-inhibited (Fig 5F, N = 4) and deeply-inhibited (Fig 5E, N = 2) units, which are located still more superficially in the EL. These units were strongly silenced as the object passed through the centers of their receptive fields.

In addition to these complex changes in response to moving objects, some units of all the different types (for example those illustrated in Fig 5B and 5E) showed a frank reduction in firing probability when the object moved from caudal to rostral. This depression consisted of relatively long periods of silence starting at about the opercular region and lasting up to the center region of the explored receptive field.

### Effects of stimulus strength on spike timing and number

In another 43 units we explored whether the effects of stepwise increments in the conductivity of a stationary object probe alter the rate and spiking patterns of the units belonging to each cluster in a similar way. The longitudinal conductance of a horizontally-oriented cylindrical object probe positioned opposite the center of the unit’s receptive field was stepped from 0.4 μS to 1 mS, (defined respectively as weak and strong stimuli), or vice versa, to provoke three-fold upward or downward increments in stimulus intensity [23].

The responses of 32 neurons could be studied systematically over more than 16 stimulus step trials separated by more than 20 s; this inter-trial interval ensured that each trial started from a relatively stationary baseline. From each trial we selected the responses to 51 consecutive EODs: EODs -25 to -1 were those occurring before the step conductance change, EOD0 was the first EOD occurring after the step change and EODs 1 to 25 were those occurring thereafter.

Stimulus conductance change steps provoked systematic changes in the number of spikes per EOD and spike-timing distribution after each EOD which were characteristic for each unit type. Post-EOD spiking patterns and their changes were first explored in superimposed rasters, aligned according to the timing of their spikes after the EOD. Fig 6 shows the altered spike timing patterns in response to upward and downward conductance steps in 2 examples of each of six different unit types. All spike timings generated within successive inter-EOD intervals are plotted as a function of the EOD sequence from EOD_-25_ to EOD_25._ For all unit types, the change in number of spikes per EOD produced when the stimulus changed from weak to strong was opposite to the change in spike number observed when the stimulus altered from strong to weak. It is particularly interesting to note that, in each of the unit types, the EOD-evoked spiking response in the presence of a stimulus object of a given strength depended on both the direction of the immediate conductance—or stimulus intensity—change and on the longer history of the presence of that stimulus object. This can be seen in Fig 6 by comparing EOD-evoked firing patterns and spike numbers in the raster zones with a gray background, corresponding to the presence of a highly conductive object, with the raster zones with no background, corresponding to the presence of weakly conductive object.

In sharp monomodal units, we observed a paradox: there is an increase in spike latency associated with a sharply organized burst of two or three spikes, thus increasing the overall mean latency (Fig 6A). An increase in spike latency associated with an increase in spike number was also observed in broad monomodal, bimodal, and trimodal units after an increase in stimulus strength. These unit types also showed a reduction in spike latency after a decrease in stimulus strength (Fig 6C, 6E and 6F).

This suggests the presence of a widely distributed early inhibition that increases and decreases with stimulus strength. Together, these recordings of different unit types indicate that transitions in stimulus intensity cause transient changes in the balance between excitation and inhibition in the early stages of sensory processing. The effects on spike number per EOD and spike timing are analyzed separately in detail below.

**Fig 7:**
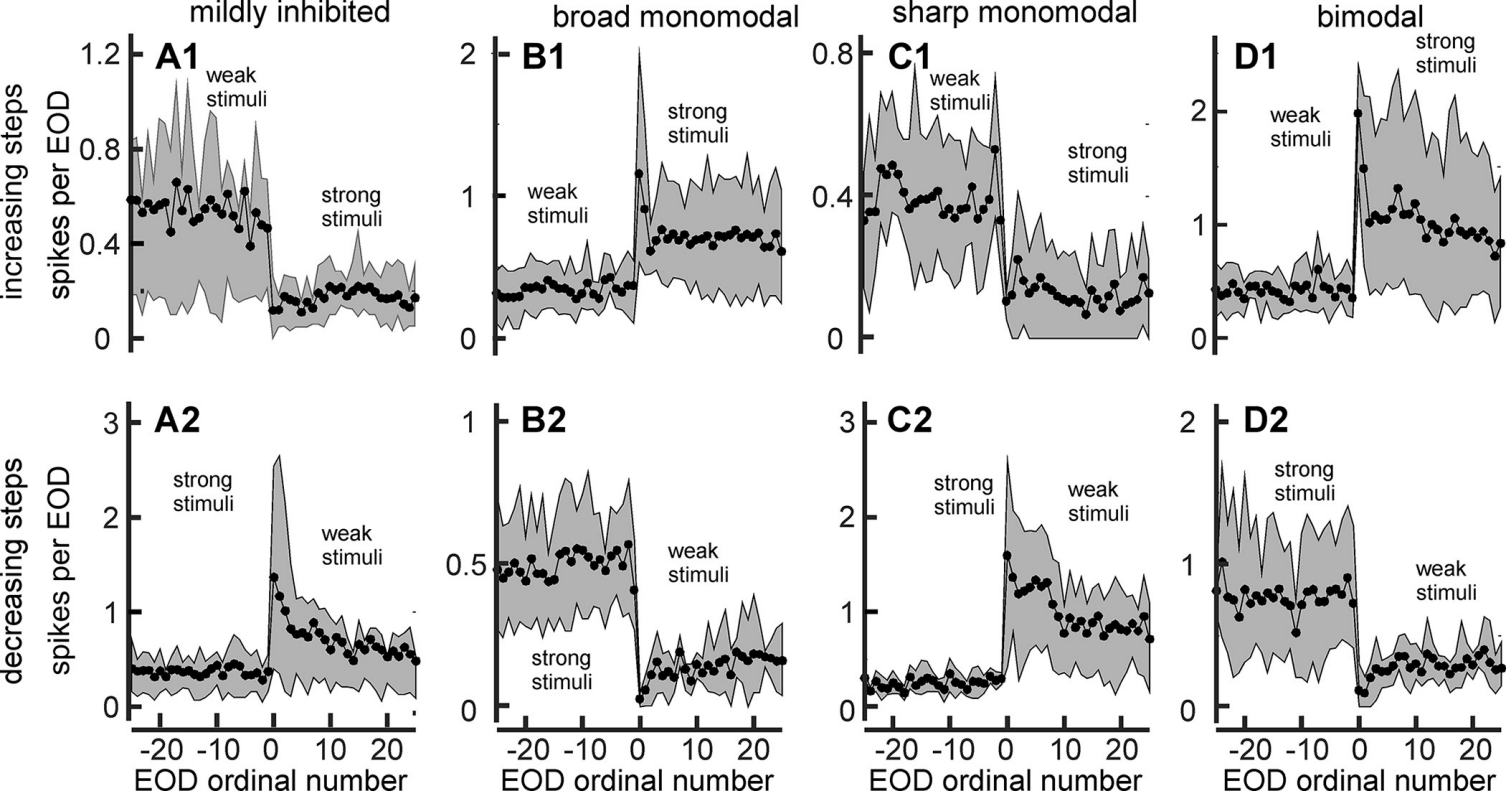
Increments in stimulus intensity provoke systematic changes in the number of spikes per EOD. The plots in the upper row show the median number of spikes evoked by each ordinal EOD (black symbols). Gray bands encompass percentiles 20 to 80. Columns correspond to different types of units (A: mildly inhibited, B: broad monomodal, C: sharp monomodal, and D: bimodal). The top row represents the effects of stepping from weak to strong stimuli (subindex 1), and the bottom row represents the effects of stepping from strong to weak stimuli (subindex 2). The first EOD after the step is marked as zero.

To evaluate the effect of a stimulus facing the center of the receptive field on the number of spikes per EOD we calculated this variable for each ordinal EOD after considering all trials in each unit. Then we calculated the mean and the standard deviation values corresponding to the units of the same type and plotted them as a function of the EOD order (Fig 7). Figs 6 and 7 show that broad monomodal, bimodal, and trimodal units increase their spike number per EOD in response to an increase in object conductance. Sharp monomodal, deeply inhibited, and mildly-inhibited units decrease their rate in response to the same change in stimulus.

To statistically compare the effects of a) stationary, b) increasing, and c) decreasing stimuli we performed an analysis of variance for each unit type using Friedman tests with repetitions.

To compare responses to weak stationary stimuli with those to strong stationary stimuli, we considered the mean unit firing rates evoked by each of the 25 EODs before a step change, in all the units of the same type. In bimodal and broad monomodal units, weak stimuli resulted in a median spike number per EOD systematically lower than those observed during strong stimuli, and can be considered “center on” units. In contrast, in mildly inhibited and sharp monomodal units in the presence of weak stimuli, spike firing rates were systematically higher than in the presence of strong stimuli, and can be considered “center off” units (Fig 7, compare baseline levels before the step). Only two trimodal and two deeply inhibited units could be explored. Their raster profiles are shown in Fig 6 B1-2 and F1-2. (S1 Table, Friedman tests)

Then we explored the responses to changes in object conductance. Friedman tests with 3 repetitions compared the mean spike number per EOD sampled for the 3 EODs just before a conductance step increment with the spike number per EOD sampled for the 3 EODs just after the change in stimulus strength. Stimulus increases provoked systematic increases in spike number per EOD in bimodal and broad monomodal units, a.nd systematic reductions in spike number per EOD in mildly inhibited and sharp monomodal units (Fig 7 and Table 1).

**Table 1:** Four distinct unit types show consistent increments in spike counts after stimulus amplitude steps.

Unit Type	Increasing step					Decreasing step				
	Median values		Friedman test 3 repetitions			Median values		Friedman test 3 repetitions		
	Pre	Post	χ^2^	DF	Pvalue value	Pre	Post	χ^2^	DF	Pvalue value
Sharp Monomodal	0.3	0.13	12.7	7, 2	3.7e-4	0.21	0.63	16	7, 2	6.3e-5
Broad Monomodal	0.34	0.66	19.0	8, 2	1.3e-5	0.46	0.05	31.7	8, 2	1.7e-8
Mildly Inhibited	0.39	0.10	11.9	6, 2	5.3e-4	0.26	1.36	15.1	6, 2	1.0e-4
Bimodal	0.37	1.4	23.7	7, 2	1.1e-6	0.5	0.07	28.1	7, 2	1.1e-7

The sample size was large enough to statistically evaluate the increments in spike counts in four types only. To evaluate whether the spike count changes systematically in the same direction after a step in stimulus amplitude, the spike counts per EOD in the 3 post-EOD pooled data distributions corresponding to the three EODs just preceding (Pre) and succeeding (Post) were compared using the Friedman test with 3 repetitions for each unit type. This procedure was repeated for increasing and decreasing steps. Median values of spike counts and Friedman test parameters are indicated for pre- and post-step increments. Significance after Bonferroni’s correction: 0.005.

The increment in spike number per EOD was phasic; this was maximal in response to the EOD_0_ and was followed by adaptation (Fig 7 and Table 2). The return to a control conditions of the spike number per EOD was faster in deeply located units when the stimulus step had caused an increase in spike number per EOD (Fig 7 C2 and D1).

**Table 2:** Four unit types show consistent adaptation in spike counts after stimulus amplitude steps.

Unit Type	Weak stimulus					Strong stimulus				
	Median values		Friedman test with 3 repetitions			Median values		Friedman test with 3 repetitions		
	Ctrl	Post	χ^2^	DF	P value	Ctrl	Post	χ^2^	DF	P value
Sharp Monomodal	0.3	0.63	18.06	7, 2	2.1e-5	0.21	0.13	9.3	7, 2	0.0024
Broad Monomodal	0.34	0.05	22.80	8, 2	1.8 e-6	0.46	0.66	10.7	8, 2	0.0011
Mildly Inhibited	0.39	1.36	7.89	6, 2	0.0050	0.26	0.10	8.9	6, 2	0.0029
Bimodal	0.37	0.07	21.47	7, 2	3.5e-6	0.5	1.4	6.6	7, 2	0.0101

The sample size was large enough to statistically evaluate the adaptation in four types only. To evaluate whether the spike counts systematically adapt after a step in stimulus amplitude, the spike counts per EOD in 3 post-EOD pooled data distributions corresponding to the same amplitude stimulus were compared: the three EODs just after a step (EOD_0_ to EOD_2_), were compared with the 3 EODs after a long-lasting steady stimulus (EOD_-3_ to EOD_-1_) using the Friedman test with 3 repetitions for each unit type. This procedure was repeated for increasing and decreasing steps. Median values of spike-counts per unit type for just after (post) and long after (control) the step and Friedman test parameters are indicated. Significance after Holm-Bonferroni’s correction: 0.05.

Spike timing distributions generated under weak or strong control (presumably steady state) stimulation conditions, before any change, were similar in most units (compare in Fig 6 the control weak stimuli, A1-F1: white background) with control strong stimuli (A2-F2: gray background). However, increments in stimulus amplitude cause important changes in post-EOD spike timing distributions, as observed in Fig 6.

**Fig 8:**
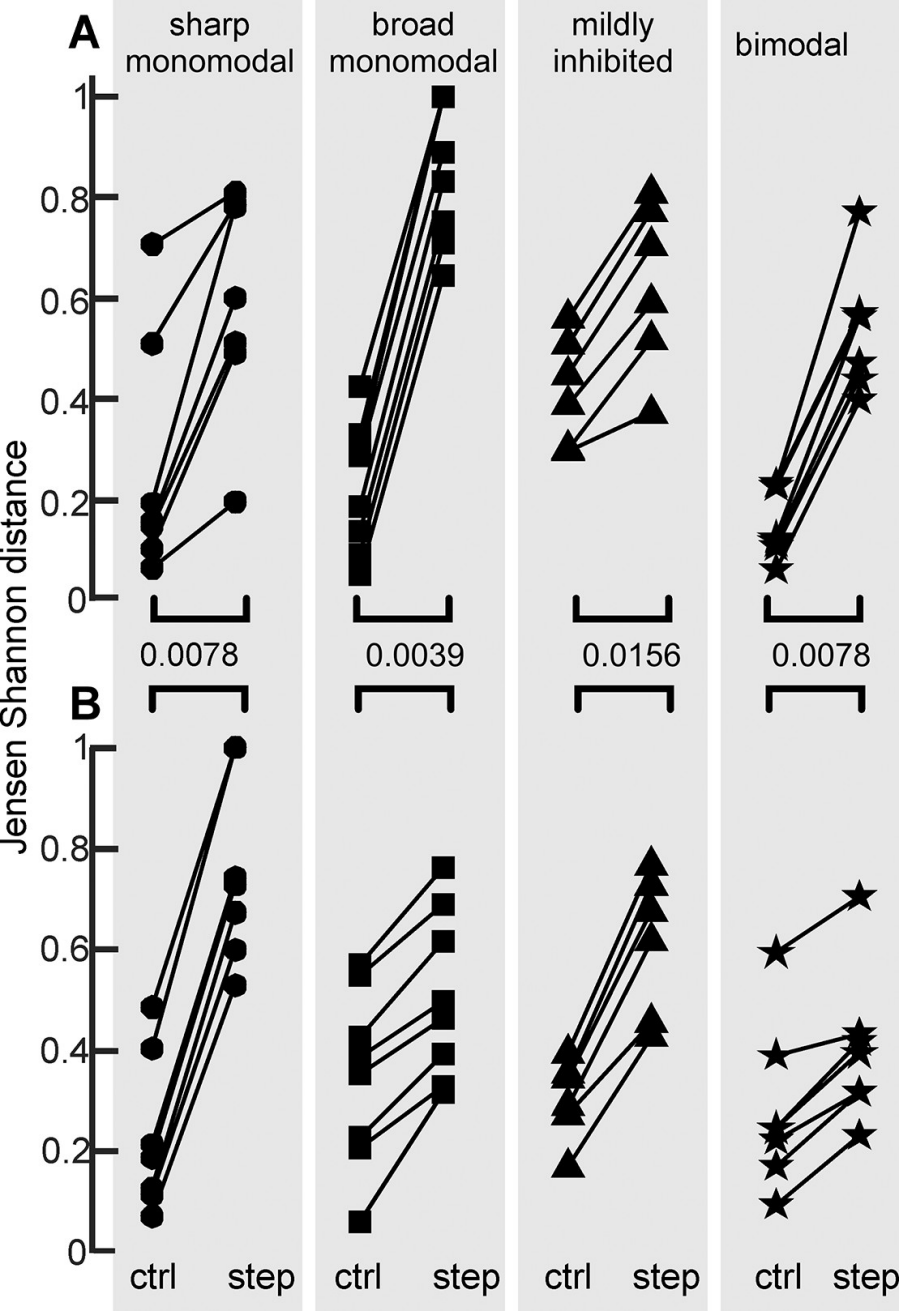
Stepwise increments in stimulus amplitude alter the information content of post-EOD spike distributions. In each plot, pairwise points linked with a line compare the differences in distributions between two pairs of triads: the left points correspond to the Jensen-Shannon divergence between control distributions (EOD_-25_ to EOD_-4_ vs. EOD_-3_ to EOD_-1_) and the right points to the Jensen-Shannon divergence between the distributions just before (EOD_-3_ to EOD_-1_) and just after (EOD_0_ to EOD_2_) the step for the same unit. Upper (A) and lower (B) rows correspond to experiments in which the stimulus was decreased and increased, respectively. To test the hypothesis that, for each unit, the step change in stimulus amplitude adds information with respect to a control condition, individual one-sided sign-rank tests were applied (p-values are indicated in between the brackets). Significance after Holm-Bonferroni adjustment for 8 comparisons: 0.05.

To evaluate the difference between spike timing distributions caused by incremental steps of the reafferent stimuli for the four unit-types in which the responses to increasing and decreasing stimulus were recorded in 6 or more experiments, we calculated the distributions of the pooled data from the responses to EODs -25 to -4 (long control before the step), to EODs -3 to -1 (control just before the step), and to EODs 0 to 2 (just after the step) and normalized them by their number of spikes. Then we used the Jensen-Shannon divergence to evaluate the differences in information in two pairs of distributions: a) just after the step and control just before the step, and b) the two controls before the step. The plots in Fig 8 show that the Jensen-Shannon divergence between the distributions just before and just after the step changes in stimulus amplitude is always larger than the Jensen-Shannon divergence between two control distributions for the same unit, for increasing and decreasing steps (Holm-Bonferroni significance level: 0.05). This indicates that the stimulus increment significantly increases the difference between pre- and post-step distributions, and therefore the change in spike timing, in itself, contains information.

**Fig 9:**
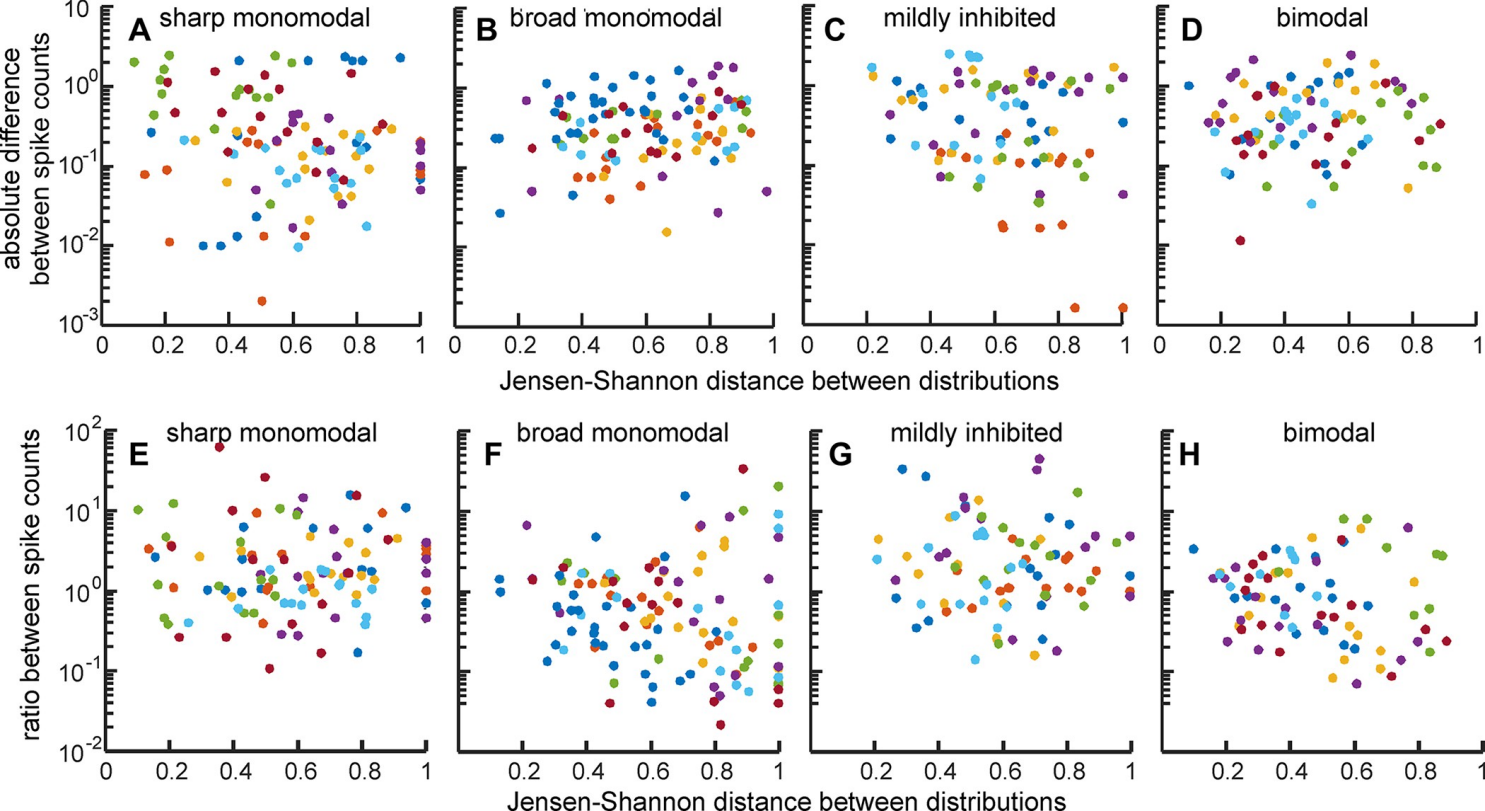
Changes in spike number and Jensen-Shannon divergence show very poor correlations. Panels A to D show the absolute difference in spike count between two distributions as a function of the Jensen-Shannon divergence between the same pairs of distributions in four unit types. Panels E to H show the ratio of spike count between two distributions as a function of the Jensen-Shannon divergence between the same pairs of distributions in the same units. Each color corresponds to a different unit in each plot. The coefficients of correlation and determination, and p values for each unit are shown in the S2 Table.

Finally, we reasoned that if the spike counts and spike timing carry information on the same features of the stimulus image, the ratio or the absolute difference in spike count and the Jensen-Shannon divergences corresponding to the same pairs of responses to the EOD should be correlated. Then for each of the four unit types, we calculated the 15 Jensen-Shannon divergences between the spike timing distributions in response to three triads of EODs (EOD_-3_ to EOD_-1_; EOD_0_ to EOD_2_; EOD_23_ to EOD_25_) for increasing and decreasing steps and correlated these with the corresponding 15 absolute differences between the mean number of spikes observed between the same triads (Fig 9A). We also correlated the Jensen-Shannon divergences between the spike timing distributions in response to the same triads with the corresponding ratios between the mean number of spikes (Fig 9B). The coefficients of determination for each unit show poor correlations (S2 Table) in both cases, suggesting that spike counts per EOD and spike timing patterns after the EOD carry complementary, non-redundant information in the same packet.

Overall, the analysis of spike firing rates indicates that broad monomodal, bimodal, and trimodal unit types are “center on” neurons and deeply, mildly inhibited, and sharp monomodal units are “center off” neurons. In addition, changes in post-EOD spike timing patterns together with the differences in unit recording sites suggest that all unit types may also use spike timing to encode changes in stimulus amplitude. This suggests that spike timing distribution after the EOD expresses the different insertion of each unit type in the circuit and that spike timing is combined with spike number per EOD forming an information packet that encodes every electrosensory image.

## Discussion

*Gymnotus omarorum* is an electric fish that explores the surroundings by emitting discrete EODs, with a waveform duration of 5 ms, separated by relatively long silences lasting about 40 ms. This provides a system for imaging the flow of information coming from a nearby environment using a regular, but dynamically variable, serial sampling regime. The main finding of this study is that electrosensory images generated by each EOD are encoded in the EL as self-organized packets. Functionally different neuron types were found in different layers of the EL and the firing behavior of these specific types has been studied individually in terms of post-EOD spiking probability distributions, focusing on both spike number and spike timing before and after the moment of EOD emission. The experiments studied the information content of reafferent sensory input signals perceived in a constant, resting environment, and evaluated changes in entropy provoked by controlled changes in the local intensity of the electric field generated by the EOD, mimicking the detection of natural stimuli. Both spike number and spike timing changed in a characteristic manner for each of the unit types identified.

Spike firing phase-locking and phase-preference after the EOD were described previously in pulse Gymnotiformes in an early report by Schlegel [39], and more recently confirmed by extracellular recordings [41]. In the present more detailed study, we show that: a) typical post-EOD firing patterns are statistically associated with a given recording location in the layered structure of the EL, and b) these patterns show typical, systematic changes in response to image alterations caused by stimulus-object movement or by impedance modulation of stationary objects. These findings suggest that each post-EOD firing pattern is characteristic for a given neuron type and informs about an underlying pattern of synaptic activity, triggered by each EOD image and elaborated by the EL circuit.

Sudden increases in conductance also cause a reduction in the immediately following inter-EOD-interval, corresponding to the well-described, fast adapting novelty response. This indicates that the information within the electrosensory lobe is encoded EOD-to-EOD and transferred, in less than 30 ms, to the electromotor pacemaker command which determines the timing of the next EOD. The experimental results thus further confirm previous theoretical predictions that novelty detection occurs early in the EL and is quickly integrated in modulation of the EOD command [53]. From this, we may conclude that every EOD-generated image is encoded by a precise avalanche of synaptic activity that progresses through the network as a single packet of activity, within which spike timing and spike numbers encode the output signal of the EL.

These results concerning neural coding in the electrosensory system motivate the following questions: a) What do the observed changes in spike-timing and spike number after the EOD in response to various stimuli suggest about the functional organization of the EL; b) How do the results support or challenge existing theories on the role of spike-timing in neural coding?

### What data suggests on the functional organization of the EL

Although the biunivocal relationship between firing patterns and anatomical neuron type has still to be confirmed by intracellular recordings, our data suggest a primary model and serve as a concrete experimental reference for guiding future studies.

The spike timing patterns of broad monomodal and two subtypes of inhibited units recorded in the superficial cell layer of EL were modulated by electrosensory stimuli, relatively late in the inter-EOD cycle. Their recording location suggests that they correspond to superficial basilar and non-basilar pyramidal neurons respectively and, consequently, that they are the output neurons of the EL that project to the torus semicircularis (red and blue in Fig 1).

Anatomically basilar neurons integrate the activity from five main inputs [21, 37, 38]. First basilar neurons are excited by primary afferents firing a burst of spikes, which synapse with the terminal branches of the basilar dendrite [21, 37, 38]. Second, basilar neurons receive a strong inhibition a few milliseconds after the onset of the afferent volley, probably coming from deep multipolar (magenta in Fig 1) and oval (not shown in Fig 1) inhibitory neurons which synapse with the descending trunk of the basilar dendrite [38, 46]. The third input to basilar neurons (not shown in Fig 1) arises from inhibitory deep granule cells and polymorphic cells which project to the somata and peri-somatic small dendrites; this projection generates surround inhibition activated from neighboring regions of the skin [21, 37, 38]. The fourth input to basilar neurons is a distributed projection originating in the granule cells of the eminentia granularis posterior, which carries a complex signal arriving via the parallel fibers of the EL molecular layer which make excitatory synaptic contacts and also drive local, molecular layer inhibitory interneurons (black lines in Fig 1). This parallel fiber input has a feedforward function that integrates input from the praeminentialis, which in turn integrates the output of bald neurons of the granule cell layer of the EL. The fifth input arrives via the stratum fibrosum which is a mixed fiber projection from the nucleus praeminentialis, carrying feedback signals from the torus semicircularis (gray arrow in Fig 1); the stratum fibrosum fibers synapse with the proximal region of the basilar neuron and non-basilar neuron apical dendritic trees.

Previously published *in vitro* recordings showed that in response to a weak intracellular current step, depolarization rises slowly accompanied by delayed spiking, probably due to a K^+^ inactivating current; stronger stimuli caused spike doublets, where the first somatic spike was followed by electrotonic activation of the dendritic tree and the generation of a dendritic spike back-propagated to the soma [42].

The responses of broad monomodal units to the EOD in the absence of objects are compatible with the intrinsic properties of basilar neurons and their insertion in the circuit (see Fig 1). Functionally, basilar neurons receive a primary afferent burst starting at about 3–5 ms after the positive peak of the EOD and lasting up to about 10–12 ms depending on the stimulus intensity [43, 44]. The occasional early spike observed in broad monomodal units may correspond to early excitation of the basilar dendritic tree by the afferent input, and the more generally present post-EOD silence lasting up to about 10 to 20 ms may correspond to inhibition originating in deep inhibitory neurons. The slow rising depolarization seen *in vitro* in basilar neurons may contribute to this silence and could also explain the low global spiking rate—only one spike fired every 2 or 3 EODs—observed in control conditions. The broad histogram of spiking behavior showing a mode about 20–25 ms after the EOD may result from a combination of the remnant excitation of the afferent burst summated together with the excitatory feedforward input to the apical dendritic tree. Both the morphology and intrinsic properties of these neurons are appropriate to their complex integrative function. The construction of the output of the EL results from the temporally structured, dynamic balance of primary afferent excitation, together with early local inhibition originating in the inner EL layers and feedforward input arriving via the apical dendritic tree.

Moving objects cause an increase in rate and a reduction of spike variability at the center of the receptive field without a reduction in spike latency. This can be explained by the increase of the afferent input and the simultaneous increase in deep local inhibition. The general depression of spiking responses to EODs after the moving object had passed through the excitatory center of the receptive field was spatially asymmetric, and depended on the direction of movement. This suggests that it is probably not primarily a result of spatially organized center-surround opposition in basilar neurons. However, looking from the point of view of time as the object moves along the body, one can consider that the effect is the lasting consequence of having previously been strongly stimulated at the center of the receptive field. This temporal phenomenon may have two possible synergistic mechanisms: a) a post-excitation plastic shift in the parallel fiber-driven balance between inhibition and excitation of the apical dendritic tree, or b) a plastically modulated increase in the activity of an inhibitory interneuron. The first of these hypotheses is also compatible with the adaptive response observed when the conductance of a static stimulus object is reduced in a stepwise manner; however, this does not preclude the possible contribution of a plastically modulated inhibitory interneuron. A good candidate for this inhibitory interneuron would be the sharp monomodal units which are inhibited by the increase in the stimulus and fire a strong burst transiently after a step reduction of the stimulus at the center of the receptive field (see Fig 6A2).

Because of their location and the inhibitory effect of the EOD, mildly and deeply inhibited units are the best candidates for non-basilar neurons. Both unit types have in common an important aversion to firing starting about 5–6 ms after the EOD and are differentiated by the strength and time course of this reduction in firing probability. Non-basilar neurons show pacemaker intrinsic properties and their firing rate follows the transmembrane voltage fluctuations smoothly in *in vitro* brain slices [42]. They receive a strong inhibitory input from multiple sources including granule neurons (green in Fig 1), polymorphic neurons (violet in Fig 1) and a complex input to the apical dendritic tree similar in origin to that of basilar neurons [21, 37, 38, 46]. Intracellular *in vitro* records show spontaneous hyperpolarizations that appear regularly at a very slow frequency. Very similar hyperpolarizing potentials can be recruited by the electrical stimulation of the EL slice either in the afferent fiber layer or in the dorsal molecular layer; this indicates that they result from synaptic inhibition [42].

Stimulation of mildly and deeply inhibited units at the center of the receptive field by increasing object conductance reduces the number of spikes per EOD and increases the duration of the firing aversion at short latencies after the EOD, confirming the inhibitory effects of the EOD. This inhibition is strong enough to cause an absolute pause in firing when a moving metal rod passes before the center of the receptive field. Mildly and deeply inhibited units were recorded in different fish but showed variable, although similar responses to the EOD. Whether these are truly different neuron types, or rather, subtypes in which changes in their responsiveness are mediated either by other inputs (e.g. serotoninergic [52]) or are season-dependent, remains unclear.

Inhibitory inputs coming from granule and polymorphic interneurons may likely account for the two waves of inhibition starting at about 5–7 and 10–12 ms observed in mildly inhibited units. These two waves of inhibition are compatible with the hypothesis that non-basilar pacemaker neurons are weakly driven in phase by the removal of an early inhibition, as occurs in other pacemaker systems [56–59]. The initial phase of this inhibition probably originates in granule neurons, and is followed either by a second activation of the same granule neurons or of another type of inhibitory interneuron. Another possibility is that ascending processes of granule cells that make gap junction contacts with the somata and somatic dendrites of non-basilar pyramidal neurons, but not with those of basilar neurons is the source of a precisely timed, intense excitation superimposed on the slower inhibitory potential. [21].

It is more difficult to identify the anatomical correspondence of the deeply located units. The principal mode of the firing distributions of bimodal, trimodal and sharp monomodal units occurs sharply about 10 ms after the EOD. These units also show faster adaptation of phasic responses to changes in electrosensory input than the more superficially located units. This suggests that primary afferent input is initially masked by the spatially distributed, deep inhibitory input originating from the multipolar and oval neurons: it is only when the primary afferent input is sufficiently strong and spatially focused, and the synaptic inhibition begins to decay, at about 10 ms, that deeply located units can fire. Moreover, when the stimulus is increased the inhibition appears more evident in the response of bimodal and trimodal neurons, causing a paradoxical association between an increase in spike latency and in the number of spikes. These three types of units are deeply located in the granule cell layer where granule neurons, polymorphic neurons and bald neurons have been described, precluding any definitive hypothesis about anatomo-functional correlation. However, in all three cases, the principal mode of their firing behaviors is well-timed either to initiate a feed forward loop with enough anticipation to pass through the nucleus praeminentialis and consequently to drive activation of parallel fibers at later latencies. In addition, their timing may be compatible to the second inhibitory wave observed in mildly and strongly inhibited neurons. However, solving this issue would have required to record pairs of units in a systematic way.

Our data show that electrosensory signal encoding in pulse Gymnotiformes differs from that observed in closely related wave Gymnotiformes, despite their very similarly organized and homologous EL. In wave-emitting fish the firing probabilities of primary afferents and EL neurons are modulated by the first and second order envelopes of the continuous sine wave-like EOD with a frequency of several hundred Hz [13–16, 20, 22], and consequently the circuit is not able to follow one-o-one the EOD cycle. It appears many of the differences between unitary responses to the self-generated, reafferent signals in wave and pulse Gymnotiformes are the consequence of a general organizational principle: similar neural networks perform according to the input regime that they receive. In other words, the structure of peripheral sensory input resulting from the animal’s exploratory strategy plays an essential role in determining how sensory signals are encoded centrally, even when they are processed by very similar neural networks.

### What role do packets of spikes have in electrosensory coding

The idea of “packet information transmission” in the brain was introduced by Kenneth Gaarder almost 60 years ago. Gaarder [2, 59] proposed that although human vision appears to be continuous for the conscious subject, the construction of a visual percept is based on three main features integrated in a single mechanism: a) discreteness of the imaging process, b) feedback control of the timing of the image by micro-saccades, and c) “packet information” storage and subtraction. In his view, retinal inputs generated by subsequent micro-saccades are stored as packets of information and cycled, saccade to saccade, in short-term memories. For each fixation point these packets share similar information for most of the points of the projected image encoded in the retina, but the small movements caused by the micro-saccades would produce signals sufficiently different to cause, after their subtraction, an enhancement of the borders and a “chiaroscuro” 3D effect [2]. The concept of information packets in neural coding, introduced by Gaarder, anticipated the basic communication technology of the present Internet. This consists of the breakdown of large units of information into smaller self-contained units that can be transmitted according to different direction tags [3]. Gaarder’s proposal also stressed the role of two other essential elements contributing to an animal’s information seeking capacity [61]: the self-generation of a reference for packet separation, and a short-term memory for packet storage and comparison.

Most animal behavior is oriented towards actively obtaining information from the environment and discreteness of image perception is typical of all active senses [60]. Reafferent inputs are caused by self-generated changes in the stimulus, that can either be shifts in the point of view (for example by eye extrinsic musculature altering gaze, or diaphragmatic strokes during sniffing), or due to pre-receptor adjustment mechanisms (for example by intrafusal fiber control by gamma motorneurons) [2, 61–63]. However, these sensory codes are optimally expressed in sensory systems in which the signal carrier is self-generated. For instance, this is the role of stroking in active touch [64–67], and electrogenesis in weakly electric fish [8, 22, 27, 68].

Our findings in *G*. *omarorum r*esemble some aspects of signal encoding in the convergently evolved, distant lineage of Mormyrinae in which the electric images are also generated by actively structured series of discrete EOD pulses [31, 68–73]. Homoplasy between African and American lineages of pulse fish is illustrated by similarity in image generation and peripheral encoding mechanisms and strategies, and in central packet information processing. In active senses where signal discreteness is self-generated, “packet” organization based on spike-timing and number requires a neural reference signal that reliably encodes action timing. In Mormyrinae this reference is an EOD-command corollary discharge [17, 19, 68]. This corollary discharge is a complex signal which preferentially gates re-afferent input to deeply located neurons, facilitates propagation to more superficially located output neurons, and is the support of a centrally generated plastic expectation, allowing the fish to suppress stagnant information or to enhance novel signals [71–74]. Although a corollary discharge from premotor regions were described in the preglomerular complex, a thalamic region exclusively connecting midbrain with pallium [75], there is no evidence of pacemaker generated corollary discharges in the EL of Gymnotiforms. This raises the question: what is the time-referencing signal in the EL pulse Gymnotiformes?

The answer may be in Mark Twain’s words referring to speech communication: “The right word may be effective, but no word was ever as effective as a rightly timed pause” [76]. Here we propose that in *G*. *omarorum* a global inhibition serves as the temporal reference and provides a strong background silence against which to detect the reafferent excitation resulting from the self-generated EOD. Present data from the pulse-emitting *G*. *omarorum*, suggests that this reference for the self-generated input may be more likely reafferent than a corollary discharge. Taking into account that the EL shows several types of inhibitory interneurons, with different potential roles in signal processing [38, 46], our data also suggest that inhibition may play a major role in constructing this reference, and is compatible with the following findings. First, our data suggests that the regular firing of the EOD that drives some of these interneurons is likely to control the pacemaker activities of non-basilar neurons. Second, trimodal and broad monomodal units fire infrequently a phase-locked single spike. This spike fires sharply at about 5 ms after the EOD at the same time that the primary afferents reach the EL. After 5 ms, there is a strong pause in spike firing. This silence is shared by sharp-monomodal, bimodal and trimodal unit types whose next spike also occurs sharply, between 10 and 12 ms. This global gating-inhibition of sharp and broad monomodal and bi- and tri-modal neurons is likely to be generated by the deep oval and multipolar GABAergic neurons scattered in the deepest layers of the EL. These cells project widely to the basilar dendrites of all cells penetrating the deep neuropil layer [21, 38, 46], rather like the GABAergic large multipolar neurons in Mormyrinae [77, 78]. Afferent bursts lasting longer than the global silence, lead to a post-inhibitory facilitation [56–58], which determines the precise recruiting of sharp monomodal, bimodal and trimodal units at about 10–12 ms after the EOD.

The intercalation of silences to separate packets is not an exceptional mechanism. Inhibition is also present in Mormyrinae in association with the corollary discharge [77, 78] and is a common feature of the auditory system in vertebrates [79–82]. “Packet” transmission, initiated and structured by silent pauses, is behaviorally crucial in speech and music perception. Structured silence is also important for localization, segregation, and grouping of sound sources [81]. An example of this type of neural encoding is found in the superior para-olivary nucleus of mammals, where octopus inhibitory neurons which selectively fire at the onset of sounds are situated in an optimal position to provide the temporal information needed for the segmentation of ongoing complex inputs into discrete events [82].

A second important feature is the ability to compare on-going input packets with stored packets. In a cerebellum like network where synaptic activity on apical dendrites is distributed in time and space over the neuron population, changes in the efficacy of time- and site- specifically activated synapses allows to store a mirror image of the packets received in the recent past [72, 83], to be compared with the present spike-timing encoded input. This was clearly shown in the EL of Mormyrinae where anti-Hebbian, spike timing dependent synaptic plasticity was first described [72, 83, 84]. This type of plasticity was also confirmed in wave Gymnotiformes and also in Rajiformes [85, 86]. Taking this last into account we have proposed that the “novelty potential” observed in the EL of pulse emitting Gymnotiformes, which occurs before the electromotor novelty response, is the consequence of the comparison between present inputs with a feed-forward driven expectation [53]. Although in general it is considered as a feedback loop [17], the rhombencephalic loop originating in the deep bald neurons of *G*. *omarorum* may be better considered as a functional feed-forward loop [53]. The early recruitment of bald neurons and the short conduction divergence through this path may allow these feed-forward signals to reach the apical dendrites of superficial neurons of the EL within the same, on-going inter-EOD interval. The activation of this apical synaptic field by the feed-forward path may then provide the background, mirror-like context necessary for the subtraction of the predicted expectation from the present sensory image [28, 51].

In summary, we propose that the central electrosensory stream is structured by silences generated by a widespread inhibition recruited by every EOD in the deeper layers of the EL where the primary afferent signal arrives. This inhibition prevents the early firing of basilar and non-basilar (pacemaker) pyramidal output neurons at the start of the inter-EOD interval and synchronizes the firing of deep neurons which project to the praeeminentialis nucleus and thus indirectly activate a functional feed-forward loop arriving to the EL via the parallel fibers of the molecular layer to synapse with the apical dendritic trees of the pyramidal neurons. This feed forward signal activates the parallel fiber synaptic field synapsing with the pyramidal neuron apical dendrites: this is the site where the packets of information generated by previous EODs have been integrated and stored, expressed in the anti-Hebbian spike timing-dependent plasticity at selected synaptic contacts. This temporal expression of a memory trace coincides with excitation of basilar pyramidal neurons and the release from inhibition of non-basilar pyramidal neurons, providing a cellular mechanism for the comparison of immediate past with present information. This serves two purposes, updating recent memory and constituting the packet of new information feeding forward to the next stage of sensory processing in the torus semicircularis. The direction or speed of goal-directed movements relative to nearby objects will change the electrosensory image differently and can be used to enhance or dilute the information about specific features. Thus, the ability to compare packets of sensory input is an essential mechanism of feature detection.

#### A code must be selectively read

The original description of packet processing of information was made for vision, a human sensory modality which appears principally designed for appositional peripheral imaging and involves complex and feedback controlled systems in charge of generating a focused “real image” on a point-to-point receptor mosaic. Responses to light-carried images are processed in the topographically organized neural network of the retinal circuit. This has suggested that border detection could be a plausible function of storage and subtraction of visual images triggered by microsaccades [2]. However, electric, auditory and lateral line senses have to deal with superposition images in which information is coming from a wide, distributed, unfocused field [87–89]. In these systems, the early stages of information representation are processed globally in cerebellum-like networks that appear to have evolved for this type of information perception [15–20].

More specifically, in the electric sense, each receptor receives the sum of distance-weighted information from a broad electrosensory receptive field extended around the whole body [9]. The sparse firing of the EL neurons, which in the absence of novel stimuli generate just one spike every two or three EODs, is appropriate for encoding this distributed image. Within the EL, in the presence of novel stimulus objects the network-wide distribution of spiking probability is restructured, with increases or decreases of spiking time and numbers in contiguous regions of the somatotopic maps. Since the information about the object is distributed across many output units of the EL, it is likely that the integration of their effects on downstream neurons depends on how spike timing in axons coming from the EL is clustered in time: the response of the next stage in the central network will be a function of both temporal and spatial convergence. This convergence provides the measure of the degree of synchrony necessary to interpret the spike timing code.

The present recordings and classification of unit activity in the EL have shown that spike timing is combined with spike number to form a packet of information in this early stage of sensory processing. These results confirm and expand previous findings which demonstrated modulation of local field potentials [24], the presence in the EL of “novelty potentials” that precede and predict the amplitude of behavioral novelty responses recorded in chronically implanted freely discharging fish [28], and the modulation of unidentified units in freely moving fish [27].

We have shown that sensory signals are organized in packets constructed from two variables that are independently modulated by a stimulus, and we have proposed a mechanism to explain packet storage and comparison by subtraction. The combination of discretization and working memory is a necessary condition for packet encoding, but a code requires to be read. The remaining challenge is to understand how a combination of the number and timing of the spikes forming the packet is read in a downstream torus neuron. Neither do we know whether single or multiple neuron types participate in reading or decoding the information contained in each packet. To demonstrate the neural sufficiency of this mechanism of information coding, i.e. to break the code, will require exploration of the responses of torus neurons to the information carried by the packets sent by pyramidal neurons.

## Conclusions

The results presented here show that both spike timing and spike number per EOD are complementary, fundamentally interactive parameters composing information packets that encode the electrosensory images generated by a series of discrete EODs. This packet encoding and comparison with a stored average of previous packets allows the EL to detect image novelties and to determine other image features. This strategy for signal encoding shows considerable similarity with that observed in the convergently evolved pulse-emitting Mormyrinae electric fish. However, in the absence of a corollary discharge mechanism to link perception to action in the pulse Gymnotiforme electrosensory system, a different mechanism consisting of a reafferent-generated silence caused by a widespread inhibition is used for separating packets in the sensory flow.

## Supporting information

S1 Table Spike counts fire differently under weak and strong steady stimulation. Friedman test with 25 repetitions contrasting the spike counts under weak and strong stimulation were calculated for each unit type in which we explored more than 5 units. Significance level after Bonferroni correction 0.00006.
(DOCX)

S2 Table Correlation coefficients, Coefficient of determination and p values (N = 15 in each case) obtained when correlating the Jensen -Shannon distance to: a) the absolute difference in spike counts and b) the ratio of spike counts, for each unit. Note that only one unit (broad monomodal #2) shows a correlation with a p_value lower than 0.1.
(DOCX)
