# Supplementary material for: Packet information encoding in a cerebellum-like circuit
Source: PLoS One. 2024 Sep 20;19(9):e0308146. doi: 10.1371/journal.pone.0308146 (PMC11414908; doi:10.1371/journal.pone.0308146)
Supplement: S1 Table — Friedman test with 25 repetitions contrasting the spike counts under weak and strong stimulation were calculated for each unit type in which we explored more than 5 units. Significance level after Bonferroni correction 0.00006. (DOCX) [file pone.0308146.s001.docx]

**S1 Table**

| unit type | χ2 | DF | p value |
| --- | --- | --- | --- |
| sharp monomodal | 19.48 | 7,2 | 1.01E-05 |
| broad monomodal | 51.63 | 8,2 | 6.71E-13 |
| mildly inhibited | 58.99 | 6,2 | 1.58E-14 |
| bimodal | 103.79 | 7,2 | 2.26E-24 |

S1 table. Spike counts fire differently under weak and strong steady stimulation. Friedman test with 25 repetitions contrasting the spike counts under weak and strong stimulation were calculated for each unit type in which we explored more than 5 units. Significance level after Bonferroni correction 0.00006.
