# Supplementary material for: Packet information encoding in a cerebellum-like circuit
Source: PLoS One. 2024 Sep 20;19(9):e0308146. doi: 10.1371/journal.pone.0308146 (PMC11414908; doi:10.1371/journal.pone.0308146)
Supplement: S2 Table — Note that only one unit (broad monomodal #2) shows a correlation with a p_value lower than 0.1. (DOCX) [file pone.0308146.s002.docx]

S2 Table

| TYPE | unit | correlation coefficient | | coefficient of determination | | P value | |
| --- | --- | --- | --- | --- | --- | --- | --- |
|  |  | difference | ratio | difference | ratio | difference | ratio |
| sharp monomodal | 1 | 0.40 | 0.30 | 0.16 | 0.09 | 0.14 | 0.28 |
|  | 2 | 0.11 | 0.11 | 0.01 | 0.01 | 0.71 | 0.69 |
|  | 3 | -0.01 | 0.11 | 0.00 | 0.01 | 0.97 | 0.71 |
|  | 4 | -0.28 | -0.17 | 0.08 | 0.03 | 0.32 | 0.55 |
|  | 5 | -0.14 | -0.07 | 0.02 | 0.00 | 0.62 | 0.81 |
|  | 6 | -0.29 | 0.12 | 0.09 | 0.01 | 0.29 | 0.68 |
|  | 7 | -0.26 | -0.21 | 0.07 | 0.04 | 0.35 | 0.46 |
| broad monomodal | 1 | 0.29 | 0.20 | 0.08 | 0.04 | 0.29 | 0.48 |
|  | 2 | 0.60 | -0.02 | 0.36 | 0.00 | 0.02 | 0.95 |
|  | 3 | 0.35 | 0.05 | 0.12 | 0.00 | 0.20 | 0.85 |
|  | 4 | 0.29 | -0.10 | 0.08 | 0.01 | 0.29 | 0.71 |
|  | 5 | 0.34 | 0.23 | 0.11 | 0.05 | 0.22 | 0.40 |
|  | 6 | 0.38 | 0.29 | 0.14 | 0.08 | 0.16 | 0.29 |
|  | 7 | 0.35 | 0.25 | 0.12 | 0.06 | 0.20 | 0.36 |
|  | 8 | 0.36 | 0.05 | 0.13 | 0.00 | 0.19 | 0.86 |
| mildly inhibited | 1 | -0.31 | -0.43 | 0.10 | 0.18 | 0.26 | 0.11 |
|  | 2 | -0.34 | 0.11 | 0.11 | 0.01 | 0.22 | 0.71 |
|  | 3 | 0.19 | NaN | 0.04 | NaN | 0.50 | NaN |
|  | 4 | 0.25 | 0.01 | 0.06 | 0.00 | 0.36 | 0.98 |
|  | 5 | -0.01 | 0.12 | 0.00 | 0.01 | 0.99 | 0.67 |
|  | 6 | -0.16 | -0.12 | 0.03 | 0.01 | 0.57 | 0.67 |
| bimodal | 1 | 0.16 | -0.28 | 0.03 | 0.08 | 0.57 | 0.32 |
|  | 2 | 0.08 | -0.25 | 0.01 | 0.06 | 0.77 | 0.38 |
|  | 3 | 0.24 | 0.01 | 0.06 | 0.00 | 0.40 | 0.98 |
|  | 4 | 0.16 | 0.23 | 0.03 | 0.06 | 0.56 | 0.40 |
|  | 5 | 0.30 | NaN | 0.09 | NaN | 0.28 | NaN |
|  | 6 | 0.24 | NaN | 0.06 | NaN | 0.39 | NaN |
|  | 7 | 0.08 | -0.25 | 0.01 | 0.06 | 0.77 | 0.38 |

S2 Table. Correlation coefficients, Coefficient of determination and p values (N=15 in each case) obtained when correlating the Jensen -Shannon distance to: a) the absolute difference in spike counts and b) the ratio of spike counts, for each unit. Note that only one unit (broad monomodal #2) shows a correlation with a p_value lower than 0.1.
